# A web-based genome-wide association study reveals the susceptibility loci of common adverse events following COVID-19 vaccination in the Japanese population

**DOI:** 10.1038/s41598-023-47632-5

**Published:** 2023-11-27

**Authors:** Shun Nogawa, Hajime Kanamori, Koichi Tokuda, Kaoru Kawafune, Miyuki Chijiiwa, Kenji Saito, Shoko Takahashi

**Affiliations:** 1 Genequest Inc., Siba 5-29-11, Minato-ku, Tokyo, 108-0014 Japan; 2 Department of Infectious Diseases, Internal Medicine, Tohoku University Graduate School of Medicine, 2-1 Seiryo-machi, Aoba-ku, Sendai, 980-8574 Japan; 3 Division of Infection Control, Tohoku University Hospital, 1-1 Seiryo-machi, Aoba-ku, Sendai, 980-8574 Japan

**Keywords:** Genetic association study, Genetic markers

## Abstract

The coronavirus disease 2019 (COVID-19) pandemic has spread rapidly worldwide. To prevent its spread, mRNA-based vaccines made by Pfizer/BioNTech (BNT162b1) and Moderna (mRNA-1273) have been widely used, including in Japan. Various adverse events have been reported following the COVID-19 mRNA vaccination, with differences observed among individuals. However, analyses of the genetic background associated with the susceptibility to side effects have been limited. In the present study, we performed genome-wide association studies (GWAS) for self-reported adverse events of the COVID-19 mRNA vaccination in 4545 Japanese individuals and identified 14 associated loci. Among these, 6p21 was associated with 37.5 °C or higher fever, 38 °C or higher fever, and muscle pain. HLA allele association analysis revealed that various HLA alleles were associated with the adverse effects; HLA-DQA1*03:01 and HLA-A*11:01 were more reliably associated with the adverse effects. Our results may enable the preparation and management of adverse effects by identifying the susceptibility to these adverse events. Furthermore, we obtained valuable data that may lead to a better understanding of the mechanisms of action of the COVID-19 mRNA vaccines.

## Introduction

The novel coronavirus disease 2019 (COVID-19), caused by severe acute respiratory syndrome coronavirus 2 (SARS-CoV-2), has spread rapidly since its emergence in December 2019 and has affected hundreds of millions of people worldwide^1^. The development and spread of safe and efficacious vaccines are expected to be the key to controlling the COVID-19 pandemic. According to the World Health Organization, several vaccines have been developed worldwide to prevent the spread of COVID-19^2^. mRNA-based vaccines made by Pfizer/BioNTech (BNT162b1) and Moderna (mRNA-1273) are among the most widely used in Japan and other parts of the world. As of November 29, 2021, the Japanese government estimated that 97.2 million people had been fully vaccinated (received two doses of either vaccine) in Japan, representing 76.7% of the country’s population^3^.

The most common side effects reported following the COVID-19 mRNA vaccination were injection site reactions, fatigue, headache, and myalgia. These side effects are reported to be mild to moderate and last for a couple of days^4^. Other local and systemic side effects of the vaccination include swelling, chills, joint pain, fever, redness, itching, nausea, diarrhea, abdominal pain, rash outside the injection site, and vomiting^4^. Serious side effects such as mild allergic reactions and anaphylaxis are rare but have been reported^5,6^. Side effects are signs of a common immune response to the vaccine and vary with age, sex, and ethnicity^7,8^. Interestingly, an elevated risk of myocarditis following the COVID-19 mRNA vaccination was observed in males aged 12–29 years^9^. *HLA* alleles have also been reported to be associated with adverse events following the COVID-19 mRNA vaccination^10,11^. However, to date, the mechanism underlying the development of side effects remains unclear, and there is still a lack of reports from non-European ethnicities.

One crucial challenge is to elucidate the factors that induce adverse events in response to the COVID-19 vaccine to understand its detailed mechanism of action. In this study, we hypothesized that individual differences in response to the COVID-19 vaccine may be explained, in part, by genetic differences. Therefore, to clarify the possible mechanisms by which host genetic variations might affect the COVID-19 vaccine treatment response, a genome-wide association study (GWAS) was performed in the Japanese population.

## Materials and methods

### Study subjects

The data were obtained through the Japanese direct-to-consumer (DTC) genetic testing services “Genequest ALL” and “Euglena MyHealth”, which are provided by Genequest Inc. (Tokyo, Japan) and Euglena Co., Ltd. (Tokyo, Japan), respectively. We asked subjects who were aged ≥ 18 years and who gave consent to participate in the study to answer internet-based questionnaires about COVID-19 vaccine adverse events. All the participants provided written informed consent for the general use of their genetic data for research. Before participating in this study, information on the study’s aim was sent to the participants and an additional study-specific agreement was obtained by opt-in. The study was conducted in accordance with the principles of the Declaration of Helsinki. The study protocol was approved by the Ethics Committees of Genequest Inc. (IRB no. 2021-0633-4) and Tohoku University Graduate School of Medicine (IRB no. 2021-1-469).

### DNA sampling, genotyping, quality control, and genotype imputation

Saliva samples were collected, stabilized, and transported using an Oragene DNA Collection Kit (DNA Genotek Inc., Ottawa, Ontario, Canada) or GeneFix Saliva DNA Collection (Cell Projects Ltd., Harrietsham, Kent, UK). Genotype analysis was performed using the Illumina Infinium Global Screening Array v1 + Customs BeadChip (Illumina, San Diego, CA, USA), which contains 704,589 markers; Infinium Global Screening Array-24 v3.0 + Customs BeadChip, which contains 655,471 markers; HumanCore-12 + Customs BeadChip, which contains 302,073 markers; HumanCore-24 + Customs BeadChip, which contains 309,725 markers; and InfiniumCore-24 + Customs BeadChip, which contains 308,500 markers. Because the analyzed single nucleotide polymorphism (SNP) sets were very different among the genotyping chips used, the subjects were divided into two groups depending on the type of genotyping chip: those analyzed using the former two chips (595,105 common markers) and those analyzed using the latter three chips (289,930 common markers). These are referred to as populations A and B, respectively. Quality control and association analysis procedures were performed separately for each cohort.

For the quality control analysis, we filtered out the SNP markers. The parameters were as follows: call rate per SNP < 0.95; Hardy–Weinberg equilibrium exact test *p*-value < 1 × 10^−6^; minor allele frequency < 0.01; SNPs not in autosomes. We also excluded subjects based on the following parameters: inconsistent sex information between the genotype and the questionnaire, call rate per subject < 0.95, closely related pairs determined using the identity-by-descent method (PI_HAT > 0.1875), and estimated non-Japanese ancestry. Quality control analyses were performed using PLINK^12,13^ (version 1.90b3.42) and Eigensoft^14^ (version 6.1.3).

Genome-wide genotype imputation was performed using a pre-phasing/imputation stepwise approach implemented in EAGLE2^15^ (version 2.4) and Minimac3^16^ (version 2.0.1). The imputation reference panel was 1000 Genomes Phase 3^17^ (version 5). Variants with low imputation quality (R^2^ < 0.3) and minor allele frequency (< 0.05) were excluded from further analyses. Finally, we used dosage data for the common 5,930,410 variants for the GWAS in populations A and B.

### Adverse events measurement

We provided internet-based questionnaires regarding the COVID-19 vaccine to the study participants. First, they were asked for the manufacturer’s name of the COVID-19 vaccine. Next, they answered questions regarding the local or systemic reactions they experienced after the first and/or second COVID-19 vaccination. The questionnaire provides 53 options of adverse events reported to occur at a frequency of 0.1% or more in the Japanese subjects^18^; the study participants selected all options that applied to themselves. For each listed adverse event, participants who selected an option were categorized as 'cases', while those who did not select it were categorized ‘controls’. Detailed information on the questionnaires is provided in Table S1.

### Genome-wide association and meta-analysis

The association between genotype dosage and the occurrence of COVID-19 vaccine adverse events was examined using a logistic regression model under the assumption of additive genetic effects. For each population, GWAS was performed, with adjustments for age and sex, using PLINK (version 2.00a3).

We combined the statistical data from both populations using a fixed-effects model and the inverse-variance weighting method with the METAL software^19^ (version 2011-03-25). Variants achieving genome-wide significance (*p* < 5.0 × 10^−8^) in the meta-analysis were considered to be associated with the occurrence of COVID-19 vaccine adverse events.

### HLA imputation and association analysis

HLA types for A, B, C, DPB1, DQA1, DQB1, and DRB1 were imputed using the HIBAG software^20,21^ with default recommendations using the HIBAG Asian reference. Posterior probabilities > 0.5 were used as genotype calls. The association between HLA type and the occurrence of COVID-19 vaccine adverse events was examined using the PyHLA software^22^ (version 1.0.0) with a logistic regression model under the assumption of additive genetic effects with adjustments for age and sex. We performed an HLA association analysis for each population and combined statistical data from both populations using a fixed-effects model and the inverse-variance weighting method with the METAL software^19^. We analyzed 95 HLA alleles at a frequency > 5%. For the multiple testing correction, HLA alleles achieving *p* < 5.26 × 10^−4^ (0.05/95) were considered to be associated with the occurrence of COVID-19 vaccine adverse events.

## Results and discussion

### Study subjects and the occurrence of COVID-19 vaccine adverse events

The participants’ characteristics are shown in Table 1. The population vaccinated with the mRNA-1273 vaccine was older and had a greater prevalence of females when compared to the population vaccinated with BNT162b1. This difference may be a result of the Japanese vaccination circumstances, where the BNT162b1 vaccine was approved first and administered to vaccination-priority targets such as elderly people and healthcare workers. The occurrence of adverse events following COVID-19 vaccination is shown in Table1 and Table S2. Systemic reactions were more prevalent after the 2nd vaccination dose than after the 1st dose (44% and 71% for BNT162b1 and 57% and 96% for mRNA-1273, for the 1st and 2nd doses, respectively). This is consistent with the results of previous studies^8,23^. Compared to the report from the Japanese Ministry of Health, Labour, and Welfare^18^, most of the adverse events had occurrences with a range of difference of ˂ 5%; 88% and 82% for the BNT162b1 vaccine, and 79% and 62% for the mRNA-1273 vaccine, for the 1st and 2nd dose, respectively (Table S2). The adverse event occurrences were very different between BNT162b1 1st dose, 2nd dose and mRNA-1273 1st dose, 2nd dose; the following analyses were performed after stratification by the kind of vaccine and the dose. Table S3–S6 showed that adverse events were correlated with each other and were not independent.

**Table 1:** Characteristics of the subjects included in the entire present study.

	BNT162b1 vaccine		mRNA-1273 vaccine	
	1st dose	2nd dose	1st dose	2nd dose
N	3024	2554	1521	1328
Female (%)	46.30	45.97	40.30	40.44
Age (mean ± SD)	52.35 ± 11.54	53.55 ± 11.49	46.00 ± 11.13	46.50 ± 11.22
Local reaction (%)				
Pain in vaccination site	86.67	82.34	86.52	80.57
Vaccination site becomes red	10.35	13.78	33.99	37.65
Swelling of vaccination site	18.78	21.34	40.37	42.02
Vaccination site becomes hard	11.71	11.82	21.04	19.73
Itching of vaccination site	8.80	11.39	26.89	26.05
Vaccination site becomes hot	17.72	17.42	37.74	38.03
Movement disorder (can’t raise the arm) at the vaccination site	23.54	19.73	30.18	25.53
Internal bleeding in vaccination site	1.39	0.78	0.99	1.20
There is nothing that applies in this	7.28	9.98	4.01	8.28
Systemic reaction (%)				
37.5 degrees Celsius or higher fever	6.58	36.22	17.75	83.21
38 degrees Celsius or higher fever	1.55	15.94	5.85	58.81
Fatigue	24.64	48.83	35.44	70.86
Chills (pathological)	2.55	11.47	6.44	37.12
Hot flash	4.83	8.89	7.30	15.44
Peripheral coolness (cold of hands and feet)	0.56	1.33	0.72	4.74
Numbness	1.65	1.53	2.76	2.48
Shivering	0.20	1.02	1.12	4.59
Dizzy	1.72	2.98	2.10	5.72
Headache	11.57	25.29	15.71	43.37
Sore throat	0.69	0.74	0.72	1.96
Mouth and throat discomfort	1.09	1.25	1.18	2.33
Back pain	1.22	4.46	2.96	9.56
Swelling of lymph nodes (especially neck, armpit, inguinal part)	0.73	2.51	2.24	1.96
Pain of lymph nodes (especially neck, armpit, inguinal part)	1.39	4.03	3.88	5.87
Chest pain	0.73	1.10	1.25	1.20
Stomach ache	0.76	1.21	0.79	1.81
Abdominal discomfort	1.22	1.41	0.79	2.11
Eye pain	0.40	0.55	0.46	1.43
Joint pain	3.64	11.55	5.85	23.64
Muscle pain	9.95	12.14	13.54	19.43
Musculoskeletal discomfort (such as muscle tension)	1.55	3.05	1.84	3.84
Limb pain	1.06	1.92	1.05	3.16
Limb discomfort	0.50	1.17	0.66	1.96
Snot	1.06	0.98	0.99	1.81
Stuffy nose	0.66	0.55	0.53	1.20
Nausea	1.65	2.47	1.97	4.52
Vomiting	0.23	0.43	0.59	1.13
Loss of appetite	1.16	4.50	2.70	14.83
Diarrhea	1.92	2.70	2.17	3.46
Loose stool	0.83	1.61	0.72	1.58
Constipation	0.23	0.39	0.33	0.23
Cough	0.60	0.43	0.59	1.58
Sneeze	0.33	0.35	0.46	0.45
Dyspnea	0.17	0.04	0.20	0.38
Muscle weakness	0.33	0.67	0.13	1.36
Stomatitis	0.40	0.63	0.26	0.98
Urticaria (rash that disappears within a few hours)	0.36	0.63	0.33	0.60
Eczema (long-term rash)	0.17	0.43	0.72	0.53
Blunt feeling	0.50	0.51	0.85	1.43
Sleepy in the daytime	5.19	8.22	4.93	9.94
Insomnia	0.43	0.82	0.66	1.28
Hyperhidrosis	0.43	0.74	0.33	1.28
There is nothing that applies in this	56.35	29.44	43.26	4.37

SD, standard deviation.

### Incidence of COVID-19 vaccine adverse events with respect to sex and age differences

Previous studies^7,23^ reported that women and younger people have a higher risk of adverse events following COVID-19 vaccination. Consistent with these studies, the present study also showed a higher risk in females (*p*-value < 0.05; 51% and 63% in BNT162b1, 39% and 53% in mRNA-1273, for the 1st and 2nd doses, respectively) and younger people (*p*-value < 0.05; 51% and 63% in BNT162b1, 31% and 41% in mRNA-1273, for the 1st and 2nd doses, respectively) (Tables S7 and S8).

### GWAS for COVID-19 vaccine adverse events

We performed a GWAS for each population and a meta-analysis of the adverse events. Due to the absence of cases in either population, we were unable to perform GWAS for the following conditions: constipation at BNT162b1 1st dose, dyspnea at BNT162b1 1st and 2nd dose and mRNA-1273 1st dose, eczema at BNT162b1 1st dose, and sneeze at mRNA-1273 1st dose. We identified 14 loci associated with the adverse events in response to the COVID-19 vaccine at the genome-wide significance level (*p*-value < 5 × 10^−8^), for the 1st or 2nd dose of the BNT162b1 or mRNA-1273 vaccines (Table 2, Table S9, Figure S1). Associations between rs9266082 and higher fever and rs13279405 and chest pain were found with a *p*-value < 0.05, for both the BNT162b1 and mRNA-1273 vaccines. However, other variants were differentially associated with the BNT162b1 and mRNA-1273 vaccines. Two hypotheses could explain this: genetic susceptibility to adverse events may differ between the two COVID-19 mRNA vaccines or the populations may have differential statistical power because of differential sample size and adverse event occurrence. In these associated loci, 6p21 (rs551634406, rs183300, rs9266082, rs375726766, rs3135408) was associated with a 37.5 °C or higher fever, 38 °C or higher fever, and muscle pain. Since 37.5 °C or higher fever and 38 °C or higher fever were correlated (Peason correlation coefficients = 0.578, Table S4), the lead variants associated with these two adverse events, despite being different, were located in the same genetic loci.

**Table 2:** Loci identified by the meta-analysis of COVID-19 vaccine adverse events.

reaction	variant	CHR	Position	EA	NEA	EAF	dose	BNT162b1 vaccine			mRNA-1273 vaccine		
								OR	SE	P	OR	SE	P
Itching at vaccination site	rs10744866	12	110104688	A	T	0.37	1st	0.89	0.12	0.317	0.55	0.11	**2.42 × 10**^**−8**^
							2nd	1.10	0.11	0.404	0.88	0.11	0.239
Movement disorder at vaccination site	rs146922515	10	23658821	CA	C	0.43	1st	1.13	0.07	0.0783	1.23	0.09	0.0286
							2nd	1.03	0.08	0.738	1.80	0.11	**3.69 × 10**^**−8**^
Internal bleeding at vaccination site	rs1217097	8	64623330	A	G	0.24	1st	4.12	0.24	**1.79 × 10**^**−9**^	1.11	0.41	0.797
							2nd	1.85	0.34	0.0699	0.25	0.78	0.0768
37.5 °C or higher fever	rs551634406	6	30965800	(A)_15_TAT	A	0.49	1st	0.90	0.11	0.354	0.96	0.11	0.703
							2nd	0.66	0.07	**1.09 × 10**^**−9**^	0.84	0.12	0.127
37.5 °C or higher fever	rs183300	6	33526951	C	T	0.46	1st	0.97	0.11	0.741	0.91	0.10	0.359
							2nd	0.66	0.06	**3.39 × 10**^**−11**^	0.82	0.11	0.0736
38 °C or higher fever	rs9266082	6	31320022	C	T	0.39	1st	1.31	0.21	0.201	1.39	0.16	0.0448
							2nd	1.60	0.08	**4.59 × 10**^**−9**^	1.13	0.09	0.144
38 °C or higher fever	rs375726766	6	33335716	C	CA	0.36	1st	1.04	0.23	0.856	0.90	0.18	0.556
							2nd	0.60	0.09	**3.67 × 10**^**−8**^	0.98	0.09	0.855
Peripheral coolness	rs10205263	2	221076324	C	T	0.090	1st	13.09	0.44	**7.30 × 10**^**−9**^	1.31	0.93	0.771
							2nd	3.61	0.41	0.00180	0.37	0.56	0.0777
Dizzy	rs67053119	8	51750913	T	A	0.075	1st	0.52	0.63	0.300	1.25	0.63	0.726
							2nd	0.93	0.38	0.851	5.84	0.32	**2.94 × 10**^**−8**^
Chest pain	rs13279405	8	12986585	T	C	0.056	1st	17.29	0.51	**2.69 × 10**^**−8**^	7.87	0.68	0.00225
							2nd	5.66	0.56	0.00210	4.38	1.02	0.146
Abdominal discomfort	rs57177321	11	33988111	T	C	0.10	1st	7.63	0.36	**2.30 × 10**^**−8**^	0.71	0.91	0.705
							2nd	1.94	0.50	0.182	0.43	0.82	0.301
Joint pain	rs34086990	8	81791475	A	AT	0.19	1st	0.72	0.31	0.284	3.78	0.24	**3.94 × 10**^**−8**^
							2nd	1.07	0.18	0.708	1.34	0.18	0.0962
Muscle pain	rs3135408	6	33275013	C	T	0.36	1st	0.82	0.09	0.0392	0.84	0.11	0.111
							2nd	0.56	0.10	**1.13 × 10**^**−8**^	0.88	0.10	0.222
Urticaria	rs2274569	1	100435079	C	T	0.064	1st	3.82	0.51	0.00811	2.94	0.79	0.170
							2nd	11.78	0.45	**3.15 × 10**^**−8**^	1.91	1.23	0.600

Loci that reached genome-wide significance after the meta-analysis following the 1st or 2nd dose of the BNT162b1 or mRNA-1273 vaccines. CHR, chromosome; EA, effect allele; NEA, non-effect allele; EAF, effect allele frequency in the entire our study subjects; OR, odds ratio of effect allele; SE, standard error for beta of effect allele; P, *p*-value.

Significant values are in bold.

The annotations of the associated variants are listed in Table 3^24,25^. The occurrence of adverse events in response to COVID-19 vaccines was previously reported to differ among ethnicities. The prevalence of fatigue as a reaction to the BNT162b2 vaccine was 59% and 69%, and fever was 16% and 38% in European and Japanese populations, respectively^8,18,23^. The prevalence of fatigue as a reaction to the mRNA-1273 vaccine was 68% and 80%, and fever was 17% and 77% in European and Japanese populations, respectively^8,18,23^. In our study, the allele frequencies of eight out of the 14 variants (rs10744866, rs146922515, rs551634406, rs183300, rs375726766, rs13279405, rs34086990, and rs3135408) differed by more than 10% between European and Japanese populations, suggesting the possibility that a difference in genetic backgrounds may influence the occurrence of adverse events in response to COVID-19 vaccines. These loci, especially 6p21, are associated with the expression of many genes, according to the Genotype Tissue-Expression (GTEx) database^25^. Therefore, these genes may influence the mechanism of action of COVID-19 vaccines. *HLA* genes’ *(HLA-B*, *C*, *DPA1*) mRNA expressions differed among the genotypes of the associated loci. *HLA* alleles have also been associated with adverse events following COVID-19 mRNA vaccination^10^. *HLA* genes have been reported to be associated with adverse events after the administration of various vaccines^26^ and drugs^27^. Increased *NOTCH4* expression in the circulating regulatory T cells of COVID-19 patients was associated with disease severity and predicted mortality^28^. The expression of *RPS18* was previously found to increase in isolated T cells upon stimulation with the live influenza virus^29^. A variant in *BAK1* and A haplotypes of *MICB* have been associated with dengue hemorrhagic fever caused by the dengue virus^30,31^. A variant in *PSORS1C1* has been associated with severe allopurinol-induced adverse reactions^32,33^. Variants in *HSP70*, *TAPBP*, and *WDR46* were found to be associated with aspirin-exacerbated respiratory disease^34–36^.

**Table 3:** Allele frequency and expression quantitative trait locus (eQTL) genes of the loci associated with the COVID-19 vaccine adverse events.

Variant	Associated reaction	Location	Allele frequency		eQTL genes
			European	Japanese	
rs10744866	Itching at vaccination site	12q24	0.20	0.37	*MVK*, *FOXN4*, *KCTD10*
rs146922515	Movement disorder at vaccination site	10p12	0.35	0.43	None in GTEx
rs1217097	Internal bleeding at vaccination site	8q12	0.28	0.24	*RP11-579E24.2*, *LINC01289*
rs551634406	37.5 °C or higher fever	6p21	1.00	0.49	None in GTEx
rs183300	37.5 °C or higher fever	6p21	0.65	0.46	*BAK1*, *COL11A2*, *DAXX*, *IP6K3*
rs9266082	38 °C or higher fever	6p21	0.30	0.39	*C4B*, *CCHCR1*, *CSNK2B*, *HCG22*, *HCG27*, *HLA-B*, *HLA-C*, *HSPA1B*, *MICB*, *MIR6891*, *NOTCH4*, *POU5F1*, *PSORS1C1*, *PSORS1C2*, *RNF5*, *SFTA2*, *TCF19*, *USP8P1*, *VARS2*, *VWA7*, *WASF5P*, *XXbac-BPG181B23.7*, *XXbac-BPG248L24.12*, *XXbac-BPG299F13.17*
rs375726766	38 °C or higher fever	6p21	0.60	0.36	None in GTEx
rs10205263	Peripheral coolness	2q35	0.071	0.09	No eQTL genes
rs67053119	Dizzy	8q11	0.12	0.075	No eQTL genes
rs13279405	Chest pain	8p22	0.24	0.056	No eQTL genes
rs57177321	Abdominal discomfort	11p13	0.072	0.1	No eQTL genes
rs34086990	Joint pain	8q21	0.042	0.19	None in GTEx
rs3135408	Muscle pain	6p21	0.54	0.36	*B3GALT4*, *BAK1*, *DAXX*, *HCG24*, *HCG25*, *HLA-DPA1*, *RGL2*, *RPS18*, *TAPBP*, *WDR46*, *ZBTB22*
rs2274569	Urticaria	1p21	0.076	0.064	*DBT*, *LRRC39*, *MFSD14A*, *RTCA*, *SASS6*, *SLC35A3*, *TRMT13*

European allele frequency, the allele frequency of European individuals (non-Finnish) in gnomAD v2,1,1; Japanese allele frequency, allele frequency in this study; eQTL genes, Variants associated with the COVID-19 vaccine adverse events were associated with the gene expression in GTEx (*p*-value < 0.0005).

### HLA association with the COVID-19 vaccine adverse events

**Figure 1:**
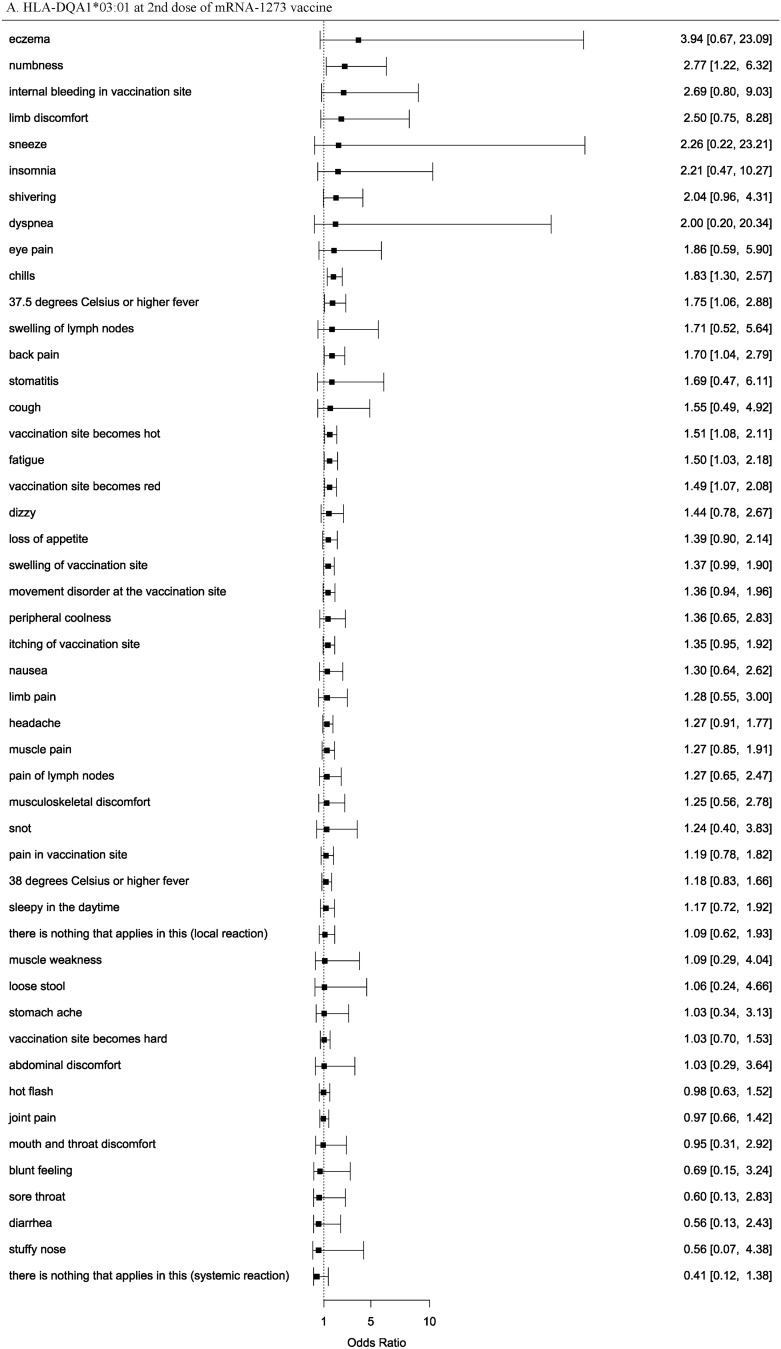

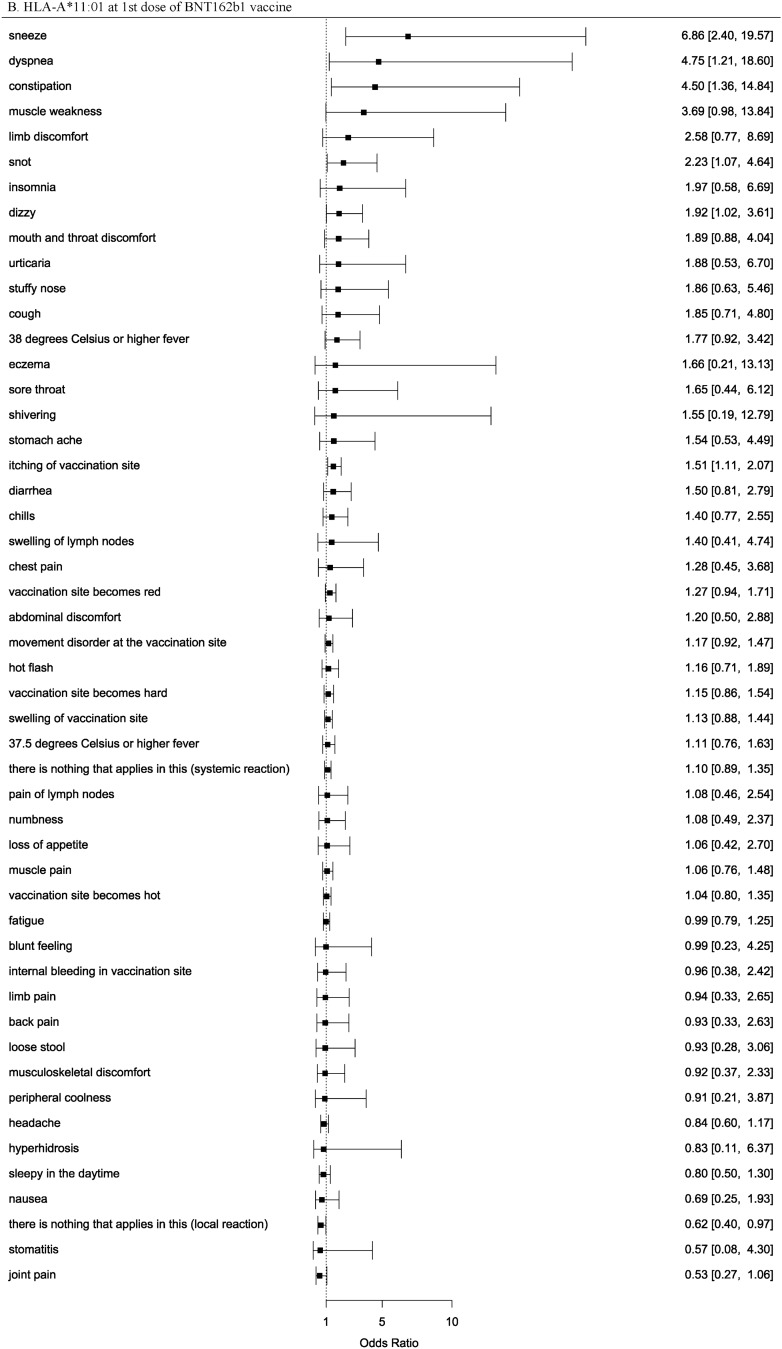
Odds ratios and 95% confidence intervals of the two reliable HLA alleles (HLA-DQA1*03:01 and HLA-A*11:01) for the occurrence of all adverse events.

We performed an HLA imputation analysis and used high quality alleles (posterior probabilities > 0.5). The frequency of HLA alleles and sample size in our data were shown in Table S10. Subsequently, we performed an HLA association analysis of the adverse events. We identified 14 HLA alleles associated with the adverse events in response to the COVID-19 vaccine at the significance level (*p*-value < 5.26 × 10^−4^), for the 1st or 2nd dose of the BNT162b1 or mRNA-1273 vaccines (Table 4, Table S11). HLA-DQA1*03:01 was associated with chills following the 2nd dose of mRNA-1273 and BNT162b1 in our study and was also reported to be associated with the adverse events of BNT162b1 in a European study^11^. This allele was considered replicated and more reliably associated. However, our study did not analyze HLA-A*03:01, the top-associated allele in a European study^11^, because of its low allele frequency (less than 0.005). As allele frequencies are far different between ethnicities, there may have been several alleles that were significantly associated with our study but were not replicated in other ethnicities. HLA-A*11:01 was associated with sneeze following the 1st dose of BNT162b1 and mRNA-1273 in our study and was also reported to be associated with severe COVID-19 in a Japanese study^37^. The risk allele was shared between the adverse events of the COVID-19 vaccine and severe COVID-19, which suggests that the allele and COVID-19 are strongly related. HLA-B*15:02 and HLA-B*15:35 were associated with adverse events in our study, and HLA-B*15:01 was associated with asymptomatic COVID-19 in a European study^38^. Therefore, HLA-B*15 might play an important role in COVID-19. However, HLA-B*15:01 was not significantly associated with adverse events in our study (Table S11). We confirmed the association between two reliable HLA alleles (HLA-DQA1*03:01 and HLA-A*11:01) and all adverse events (Fig. 1, Figure S2). The risk for several adverse events were similarly elevated; however, due to the low frequency of the HLA alleles and the infrequent occurrence of adverse events, the associations between the two HLA allele and adverse events had wide confidence intervals and were not statistically significant. To examine whether associated HLA alleles and variants were correlated, we analyzed the correlation coefficients between HLA alleles and variants (Table S12). As a result, these variants and DPB1, DQA1, DQB1 alleles were moderately correlated and the top correlation coefficient was -0.461 between rs375726766 and DPB1*09:01.

**Table 4:** HLA alleles identified by the HLA association analysis of the COVID-19 vaccine adverse events.

Reaction	HLA allele	Dose	BNT162b1 vaccine			mRNA-1273 vaccine		
			AF	OR	*P*	AF	OR	*P*
Pain in vaccination site	DQA1*01:01	1st	0.1	1.04	0.917	0.09	3.58	**0.0002932**
		2nd	0.09	1.72	0.2394	0.09	1.22	0.3588
Pain in vaccination site	DQB1*05:01	1st	0.1	1.15	0.3469	0.08	3.05	**0.000443**
		2nd	0.06	1.27	0.1741	0.08	1.33	0.1802
Swelling of lymph nodes (especially neck, armpit, inguinal part)	B*15:02	1st	0.12	4.35	**0.00008469**	NA	NA	NA
		2nd	NA	NA	NA	NA	NA	NA
37.5 °C or higher fever	DPB1*09:01	1st	0.15	0.84	0.383	0.1	0.97	0.8852
		2nd	0.15	0.65	**0.0003488**	0.1	1.04	0.8483
37.5 °C or higher fever	B*40:06	1st	0.07	1.03	0.8866	0.06	1.22	0.4482
		2nd	0.13	0.67	**0.0002165**	0.06	0.84	0.5695
37.5 °C or higher fever	DQB1*06:01	1st	0.19	1.11	0.7392	0.21	0.87	0.2867
		2nd	0.13	0.62	**0.000009773**	0.21	0.9	0.4149
37.5 °C or higher fever	DRB1*11:01	1st	0.07	0.52	0.04899	NA	NA	NA
		2nd	0.14	0.63	**0.0001659**	NA	NA	NA
37.5 °C or higher fever	DQA1*06:01	1st	NA	NA	NA	NA	NA	NA
		2nd	0.2	0.7	**0.00007333**	NA	NA	NA
Muscle pain	DRB1*11:01	1st	0.07	0.79	0.3191	NA	NA	NA
		2nd	0.14	0.47	**0.0002821**	NA	NA	NA
Limb pain	DQA1*05:03	1st	NA	NA	NA	NA	NA	NA
		2nd	0.07	3.27	**0.0004283**	NA	NA	NA
Sneeze	A*11:01	1st	0.08	6.86	**0.0003181**	0.09	5.53	0.0124
		2nd	0.11	1.53	0.5975	0.09	1.38	0.7743
Muscle weakness	DQB1*03:01	1st	0.1	1.85	0.5959	0.12	5.95	0.1866
		2nd	0.08	1.13	0.8376	0.11	4.42	**0.0001313**
Sleepy in the daytime	B*15:35	1st	NA	NA	NA	NA	NA	NA
		2nd	0.12	1.74	**0.0003864**	NA	NA	NA
There is nothing that applies in this (systemic reaction)	DQA1*01:02	1st	0.21	1.57	0.05491	0.19	1.7	**0.000006869**
		2nd	0.21	1	0.9944	0.18	1.09	0.7831
Chills (pathological)	DQA1*03:01	1st	0.15	0.95	0.8494	0.1	0.9	0.7486
		2nd	0.12	1.39	0.02235	0.1	1.83	**0.0005086**

HLA alleles achieving *p* < 5.26 × 10^−4^ (0.05/95) at the 1st or 2nd dose of the BNT162b1 or mRNA-1273 vaccines.

AF, allele frequency; OR, odds ratio of allele; P, *p*-value.

Significant values are in bold.

### Limitations

This study had some limitations. Initially, 53 types of adverse events were included; however, multiple test corrections were not performed because the adverse events were correlated. Therefore, the associated loci that we identified could include false positives, and replicate studies are required. Second, our data on the adverse events were based on web-based self-reports and might have been affected by recall bias. Third, our data might include subjects who responded to the questionnaire before experiencing any adverse events. However, the occurrence of adverse events was similar to that reported in a large-scale survey performed in Japan^18^. Thus, our adverse event data are considered reliable. Finally, our GWAS was based only on the Japanese population. Therefore, our results may not be directly applicable to individuals of other ethnicities.

## Conclusions

In this study, we performed GWAS for adverse events following the COVID-19 vaccination. To the best of our knowledge, this study is the first of its kind to focus on East Asian populations. We identified 14 loci associated with the adverse effects of the COVID-19 vaccines in the Japanese population. HLA allele association analysis revealed that various HLA alleles were associated with these adverse effects; HLA-DQA1*03:01 and HLA-A*11:01 were reliably associated with the adverse events. We discovered that genetic background is associated with the susceptibility to experiencing adverse events following the COVID-19 vaccination. Our results may enable the preparation and management of the occurrence of adverse events based on their susceptibility. Furthermore, we obtained valuable basic data that can be used to investigate the mechanisms of action of COVID-19 vaccines.

### Supplementary Information

Supplementary Figures.

Supplementary Tables.

## Data Availability

The published article and its additional files include all the data analyzed in this study. The summary statistics of the imputed GWASes were deposited on Zenodo (https://doi.org/10.5281/zenodo.8381695). All other data are available from the corresponding author upon reasonable request.
