# Supplementary material for: A web-based genome-wide association study reveals the susceptibility loci of common adverse events following COVID-19 vaccination in the Japanese population
Source: Sci Rep. 2023 Nov 27;13:20820. doi: 10.1038/s41598-023-47632-5 (PMC10682012; doi:10.1038/s41598-023-47632-5)
Supplement: Supplementary file 1 — Supplementary Figures. [file 41598_2023_47632_MOESM1_ESM.pdf]

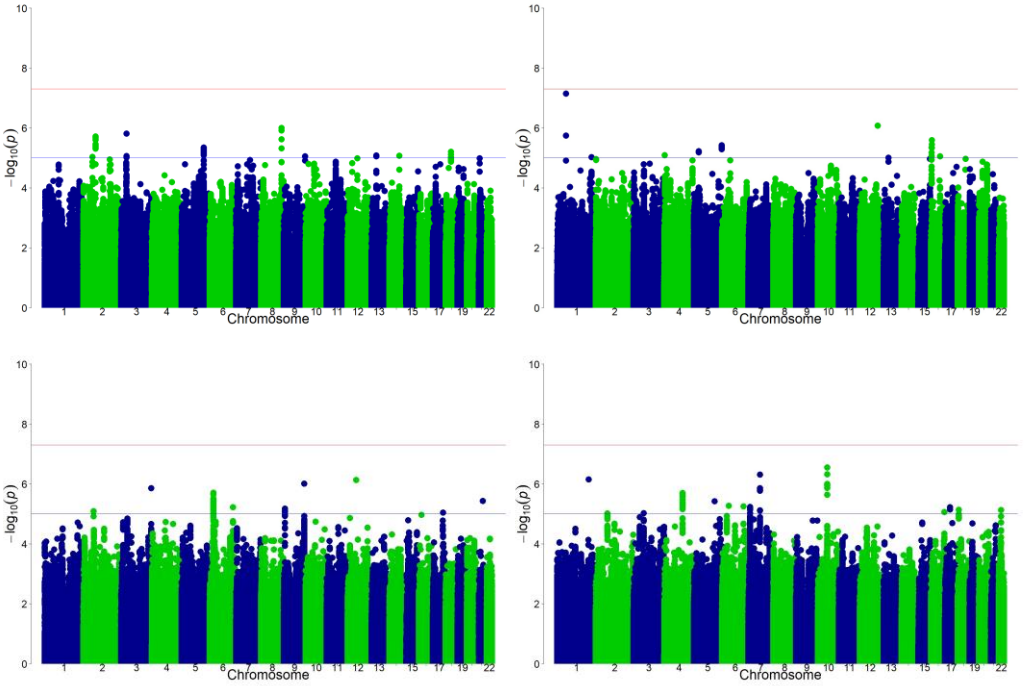

GWAS manhattan plots for pain in vaccination site

top left: BNT162b1 1st dose, top right: BNT162b1 2nd dose,

bottom left: mRNA-1273 1st dose, bottom right: mRNA-1273 2nd dose

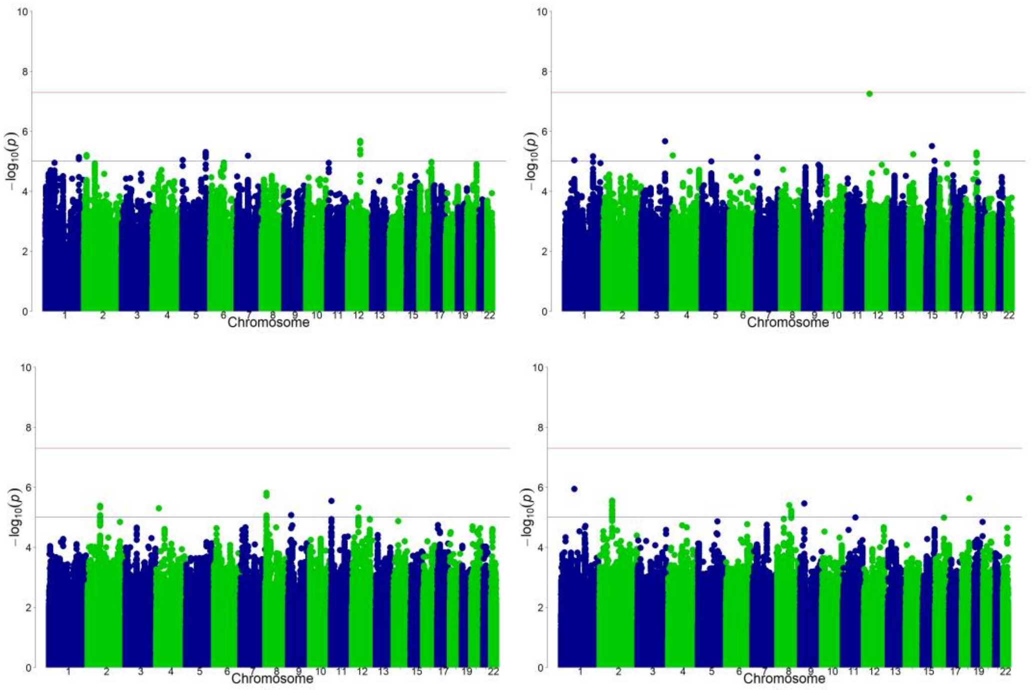

GWAS manhattan plot for vaccination site becomes red

top left: BNT162b1 1st dose, top right: BNT162b1 2nd dose,

bottom left: mRNA-1273 1st dose, bottom right: mRNA-1273 2nd dose

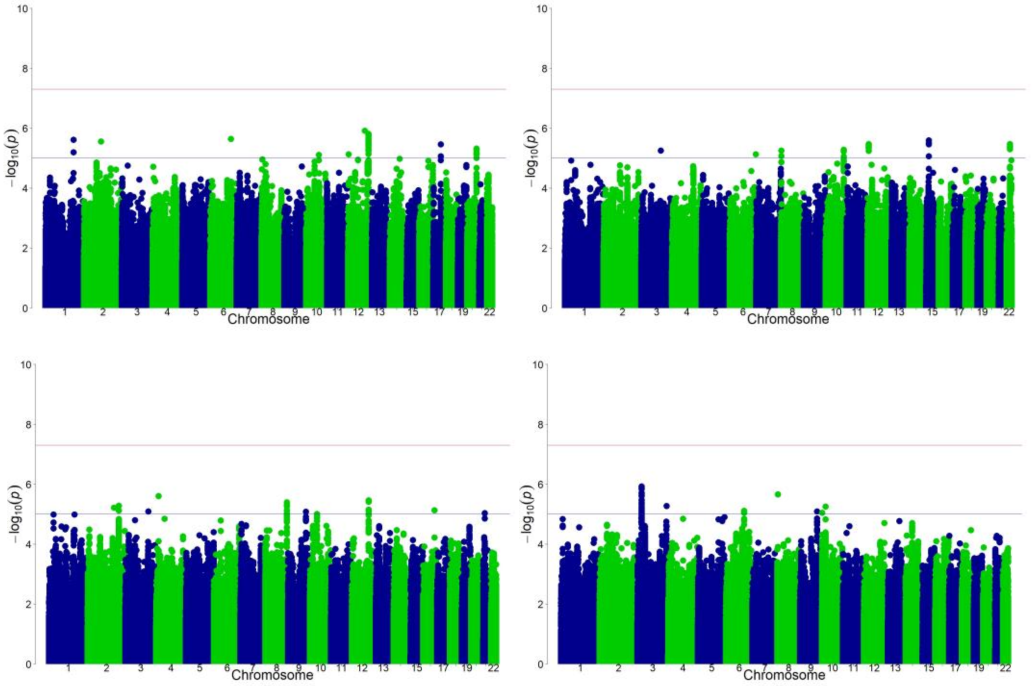

GWAS manhattan plot for swelling of vaccination site

top left: BNT162b1 1st dose, top right: BNT162b1 2nd dose,

bottom left: mRNA-1273 1st dose, bottom right: mRNA-1273 2nd dose

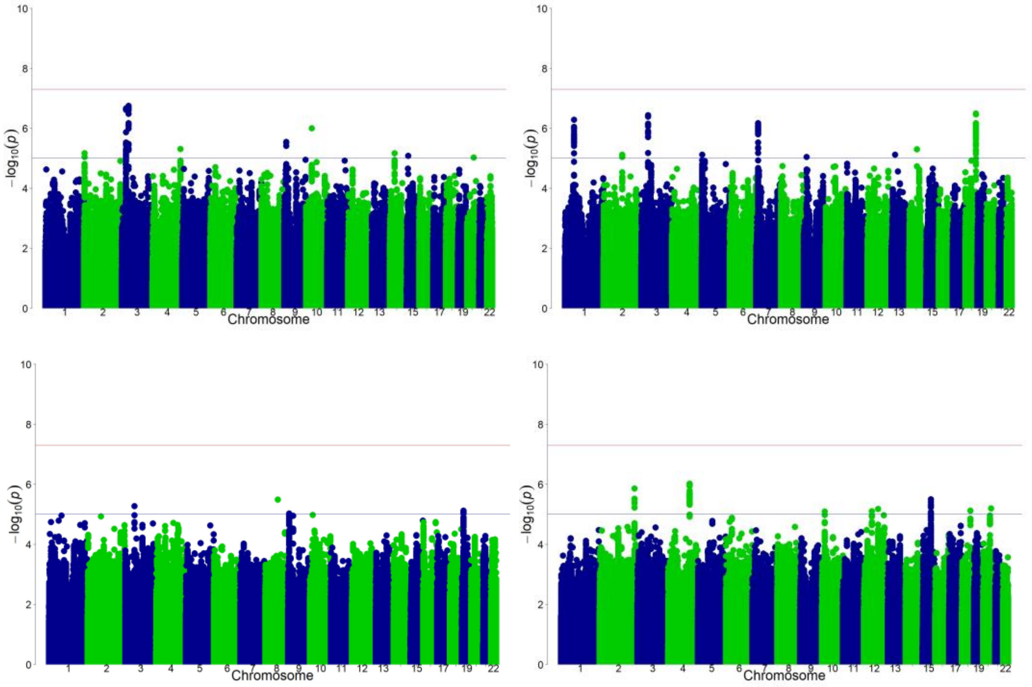

GWAS manhattan plot for vaccination site becomes hard

top left: BNT162b1 1st dose, top right: BNT162b1 2nd dose,

bottom left: mRNA-1273 1st dose, bottom right: mRNA-1273 2nd dose

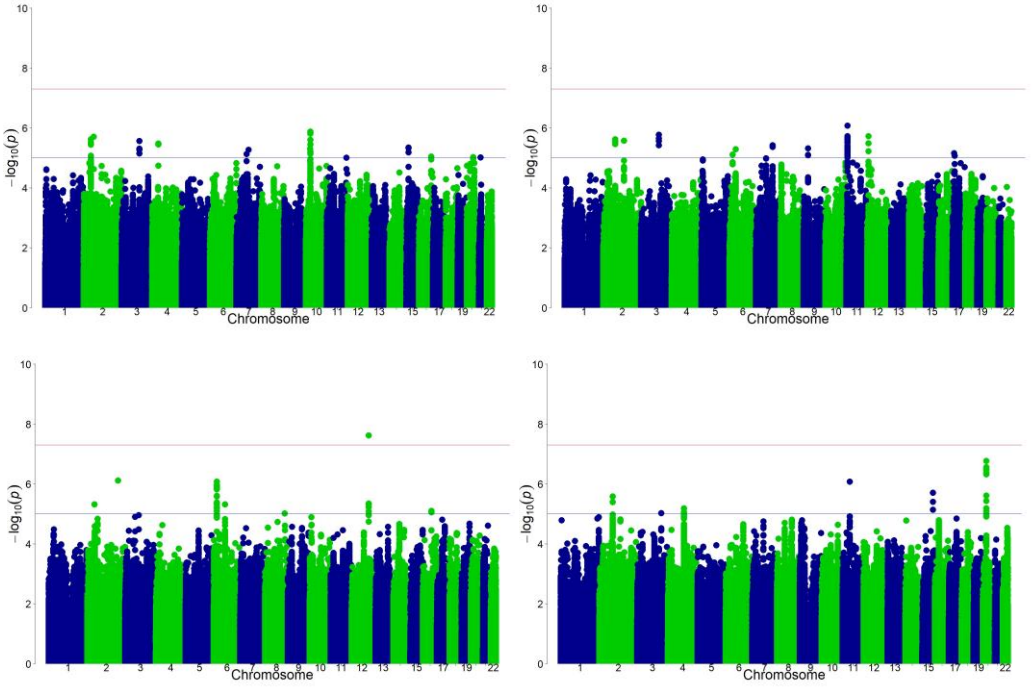

GWAS manhattan plot for itching of vaccination site

top left: BNT162b1 1st dose, top right: BNT162b1 2nd dose,

bottom left: mRNA-1273 1st dose, bottom right: mRNA-1273 2nd dose

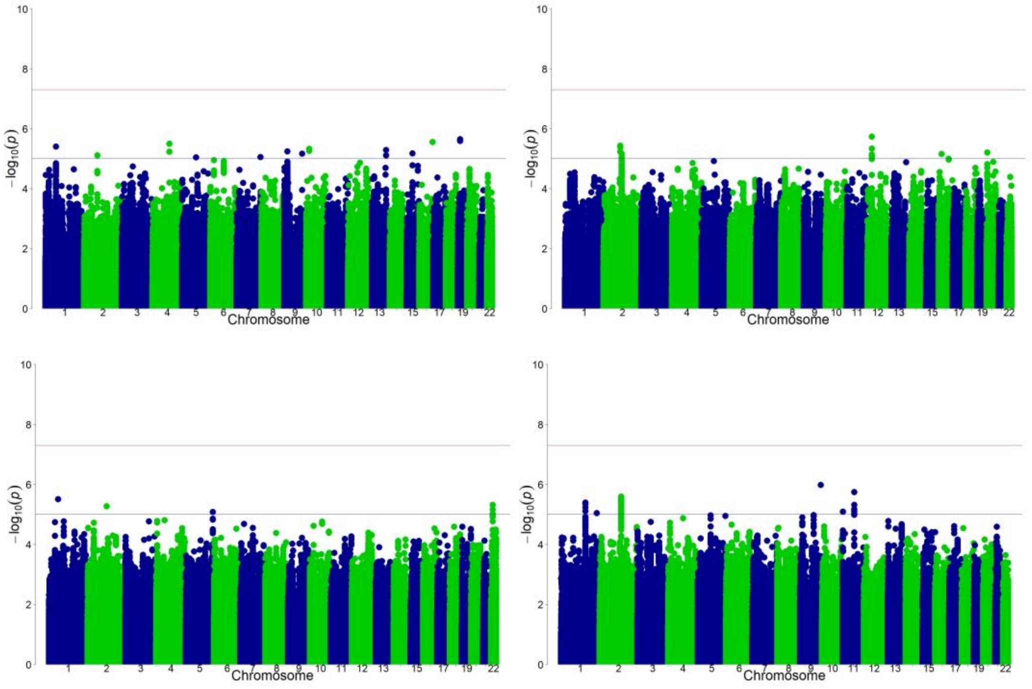

GWAS manhattan plot for vaccination site becomes hot

top left: BNT162b1 1st dose, top right: BNT162b1 2nd dose,

bottom left: mRNA-1273 1st dose, bottom right: mRNA-1273 2nd dose

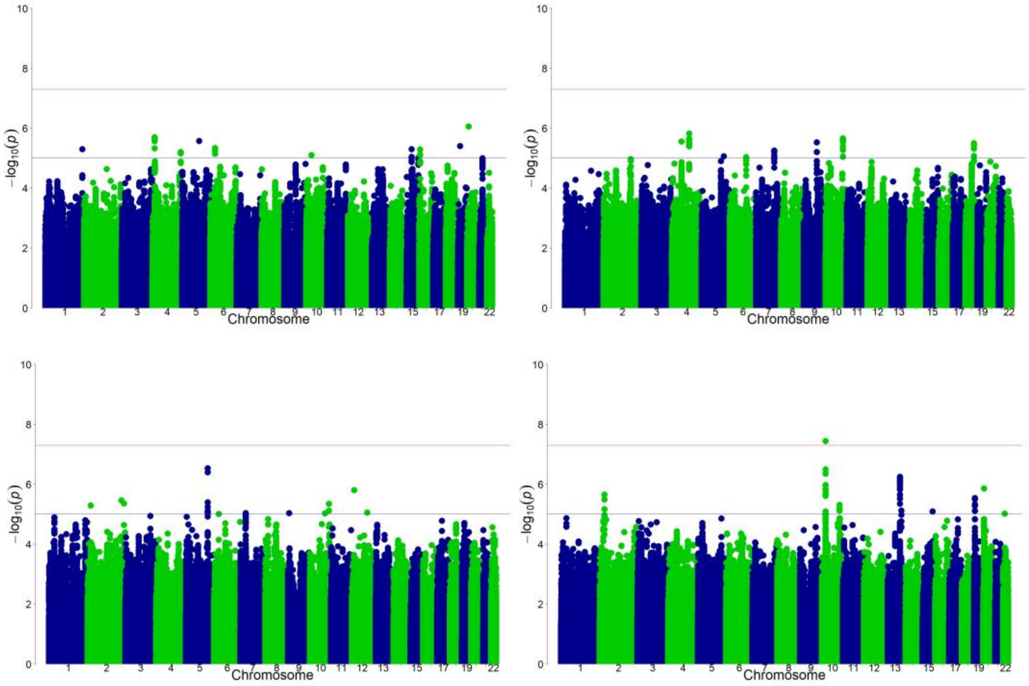

GWAS manhattan plot for movement disorder (can't raise the arm)
top left: BNT162b1 1st dose, top right: BNT162b1 2nd dose,
bottom left: mRNA-1273 1st dose, bottom right: mRNA-1273 2nd dose

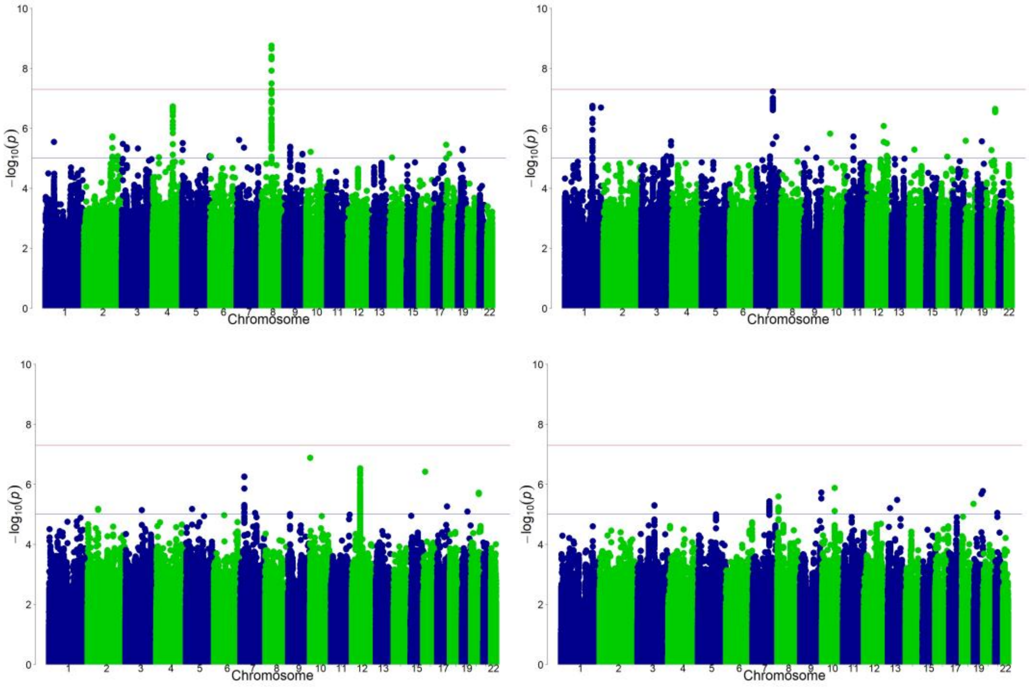

GWAS manhattan plot for internal bleeding in vaccination site
top left: BNT162b1 1st dose, top right: BNT162b1 2nd dose,
bottom left: mRNA-1273 1st dose, bottom right: mRNA-1273 2nd dose

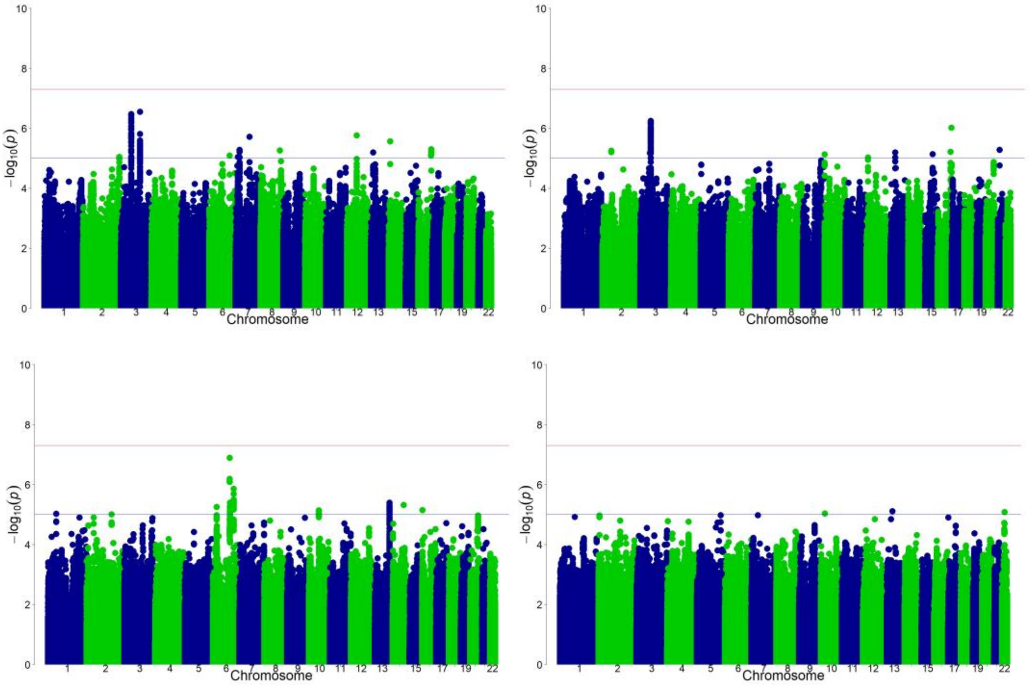

GWAS manhattan plot for there is nothing that applies in this (local reaction)

top left: BNT162b1 1st dose, top right: BNT162b1 2nd dose,

bottom left: mRNA-1273 1st dose, bottom right: mRNA-1273 2nd dose

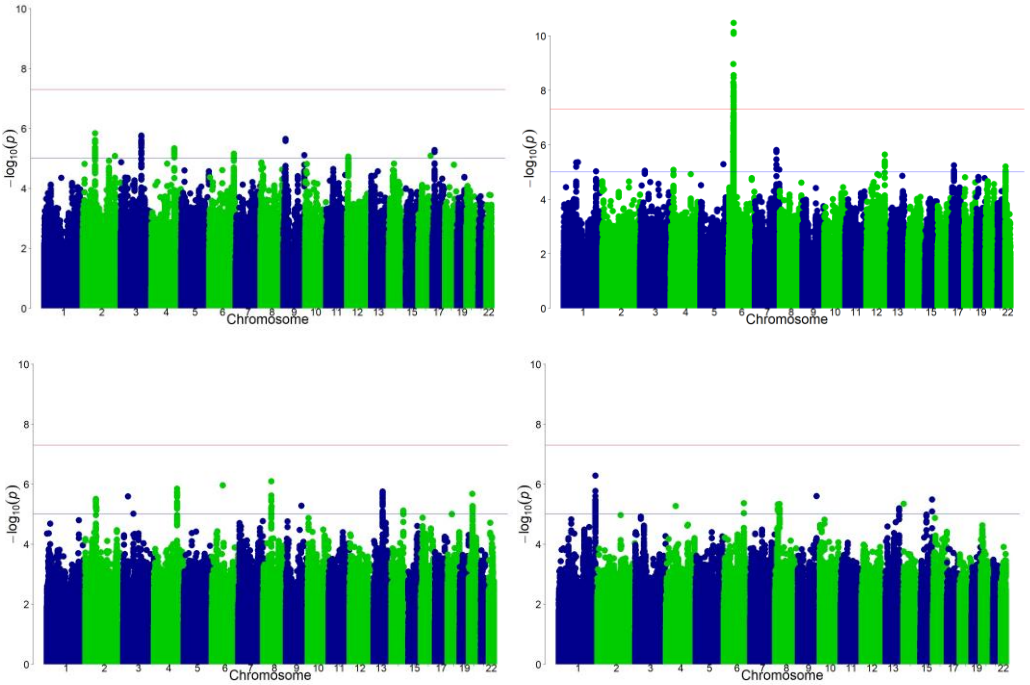

GWAS manhattan plot for 37.5 degrees Celsius or higher fever

top left: BNT162b1 1st dose, top right: BNT162b1 2nd dose,

bottom left: mRNA-1273 1st dose, bottom right: mRNA-1273 2nd dose

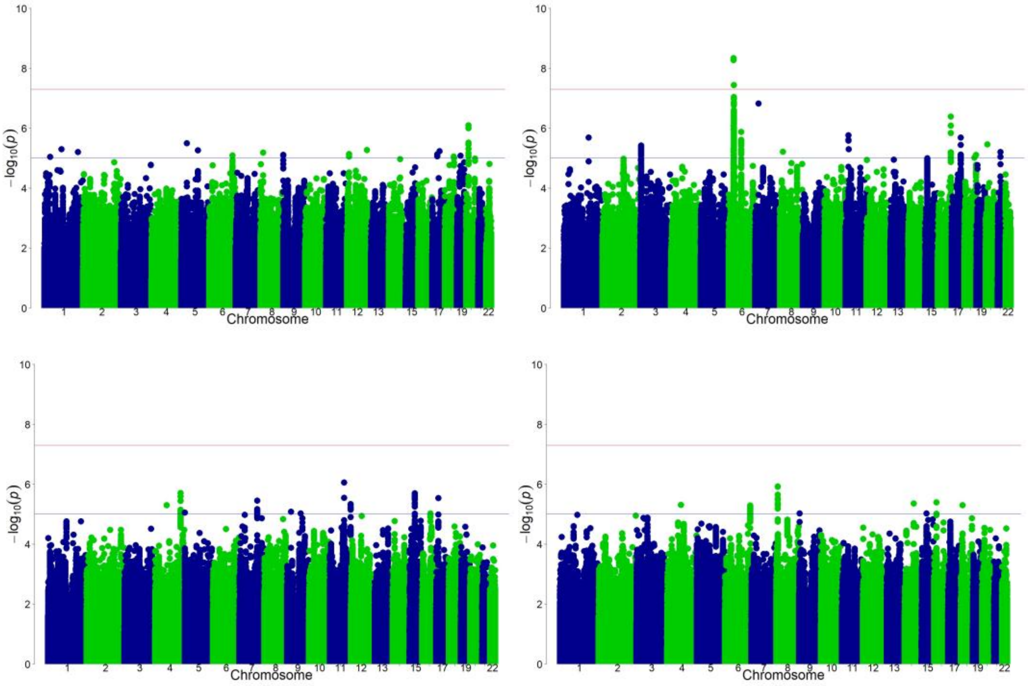

GWAS manhattan plot for 38 degrees Celsius or higher fever

top left: BNT162b1 1st dose, top right: BNT162b1 2nd dose,

bottom left: mRNA-1273 1st dose, bottom right: mRNA-1273 2nd dose

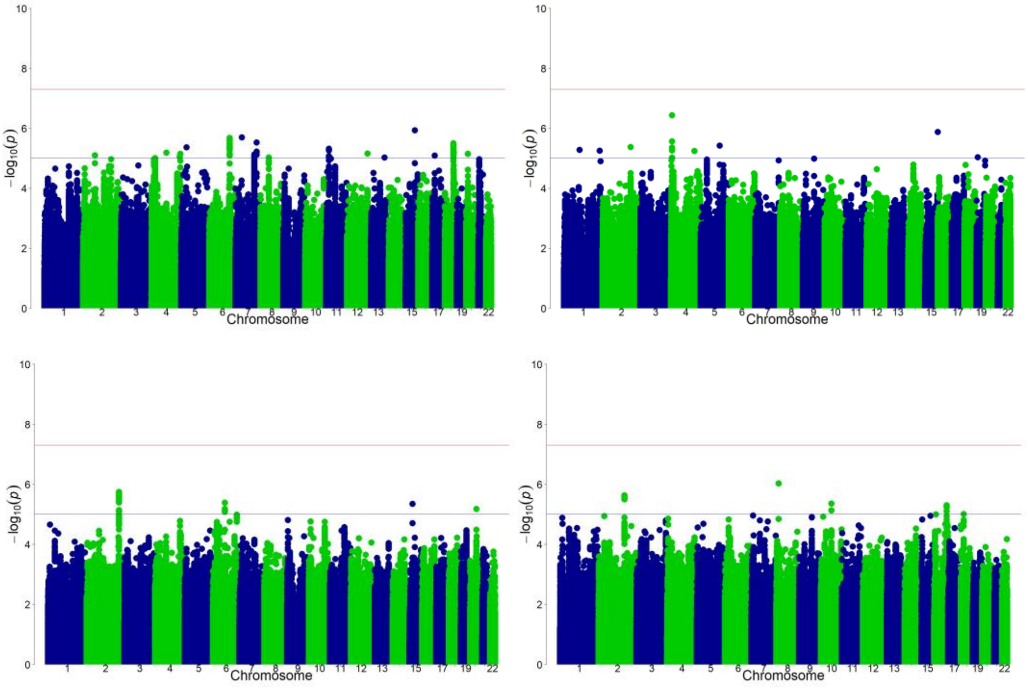

GWAS manhattan plot for fatigue

top left: BNT162b1 1st dose, top right: BNT162b1 2nd dose,

bottom left: mRNA-1273 1st dose, bottom right: mRNA-1273 2nd dose

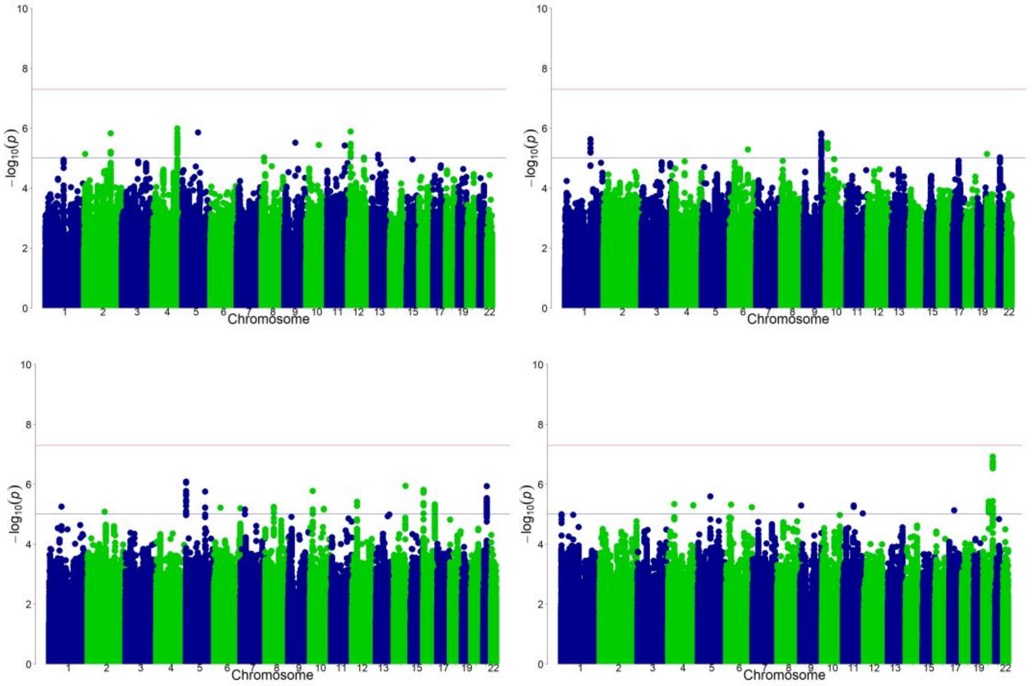

GWAS manhattan plot for chills (pathological)

top left: BNT162b1 1st dose, top right: BNT162b1 2nd dose,

bottom left: mRNA-1273 1st dose, bottom right: mRNA-1273 2nd dose

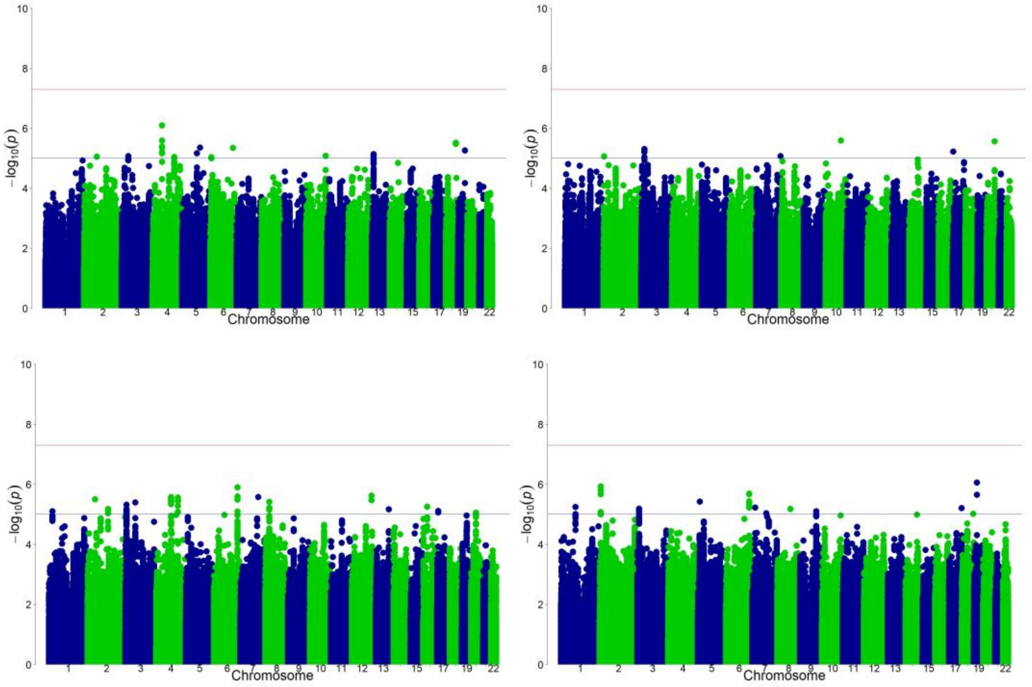

GWAS manhattan plot for hot flash

top left: BNT162b1 1st dose, top right: BNT162b1 2nd dose,

bottom left: mRNA-1273 1st dose, bottom right: mRNA-1273 2nd dose

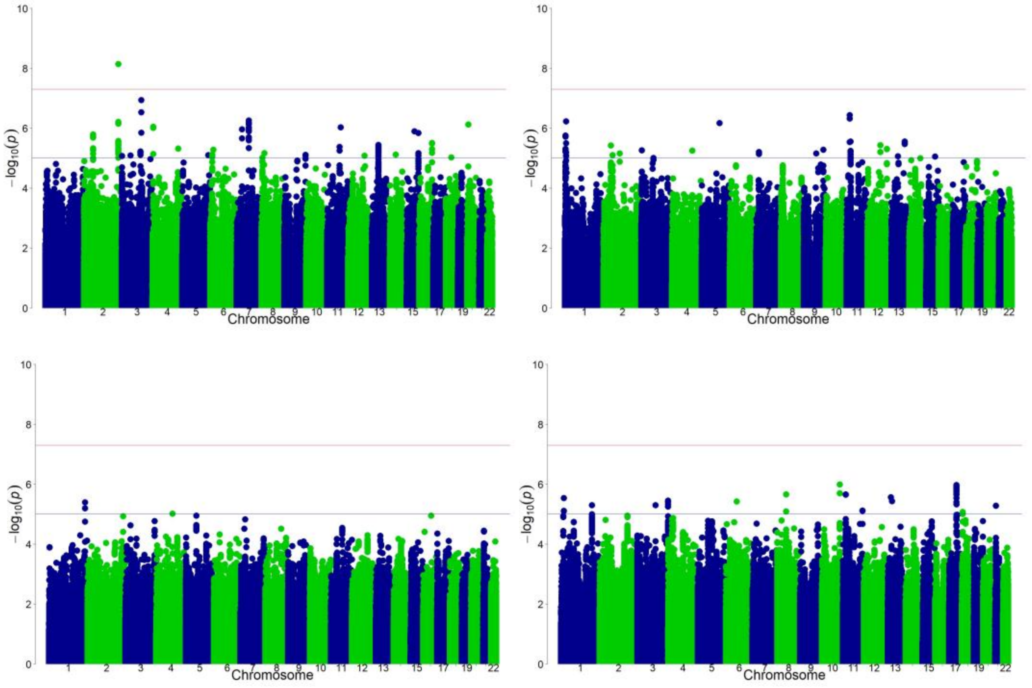

GWAS manhattan plot for peripheral coolness (cold of hands and feet)

top left: BNT162b1 1st dose, top right: BNT162b1 2nd dose,

bottom left: mRNA-1273 1st dose, bottom right: mRNA-1273 2nd dose

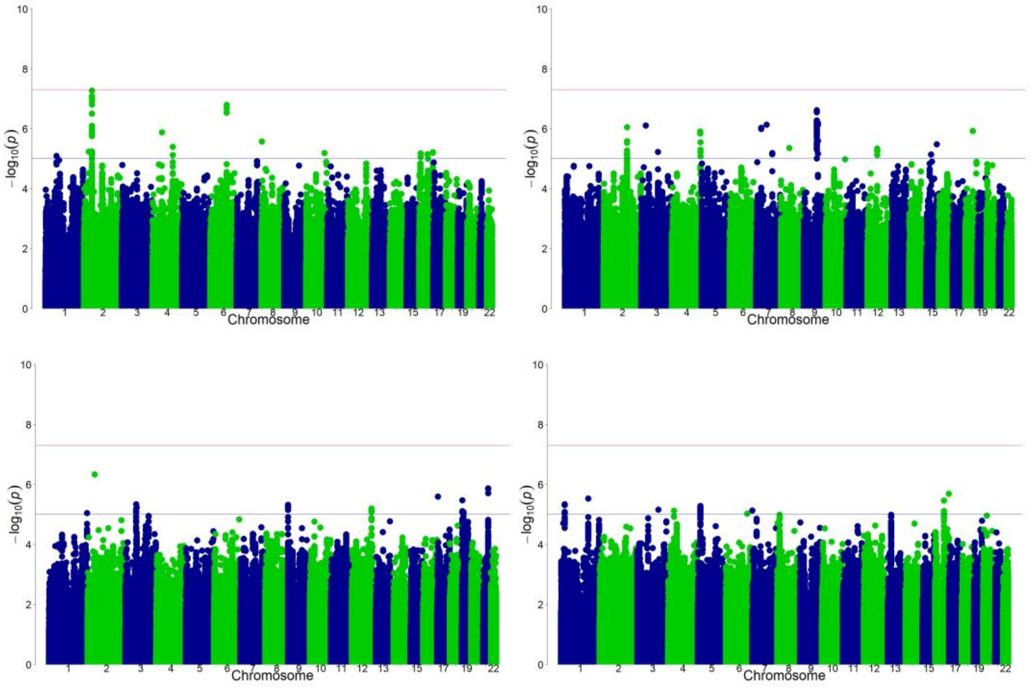

GWAS manhattan plot for numbness

top left: BNT162b1 1st dose, top right: BNT162b1 2nd dose,

bottom left: mRNA-1273 1st dose, bottom right: mRNA-1273 2nd dose

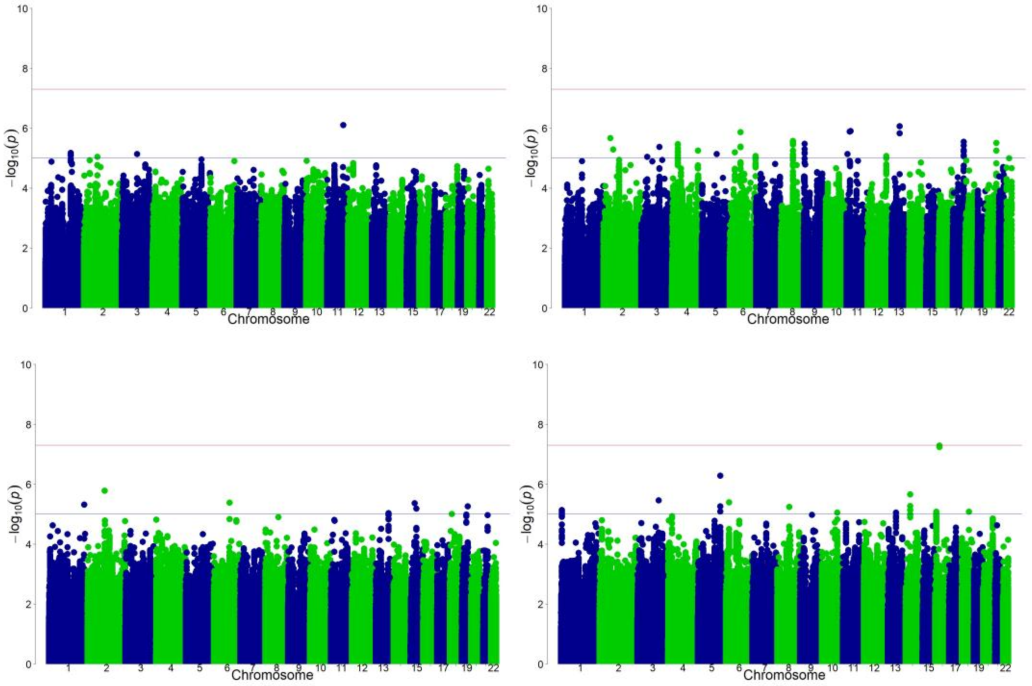

GWAS manhattan plot for shivering

top left: BNT162b1 1st dose, top right: BNT162b1 2nd dose,

bottom left: mRNA-1273 1st dose, bottom right: mRNA-1273 2nd dose

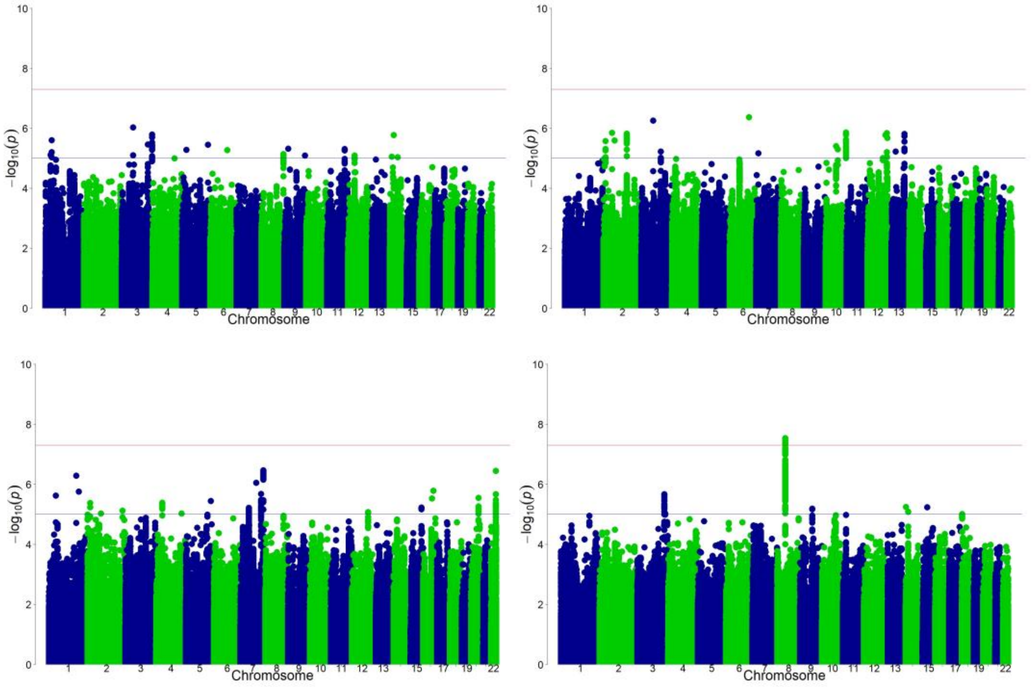

GWAS manhattan plot for dizzy

top left: BNT162b1 1st dose, top right: BNT162b1 2nd dose,

bottom left: mRNA-1273 1st dose, bottom right: mRNA-1273 2nd dose

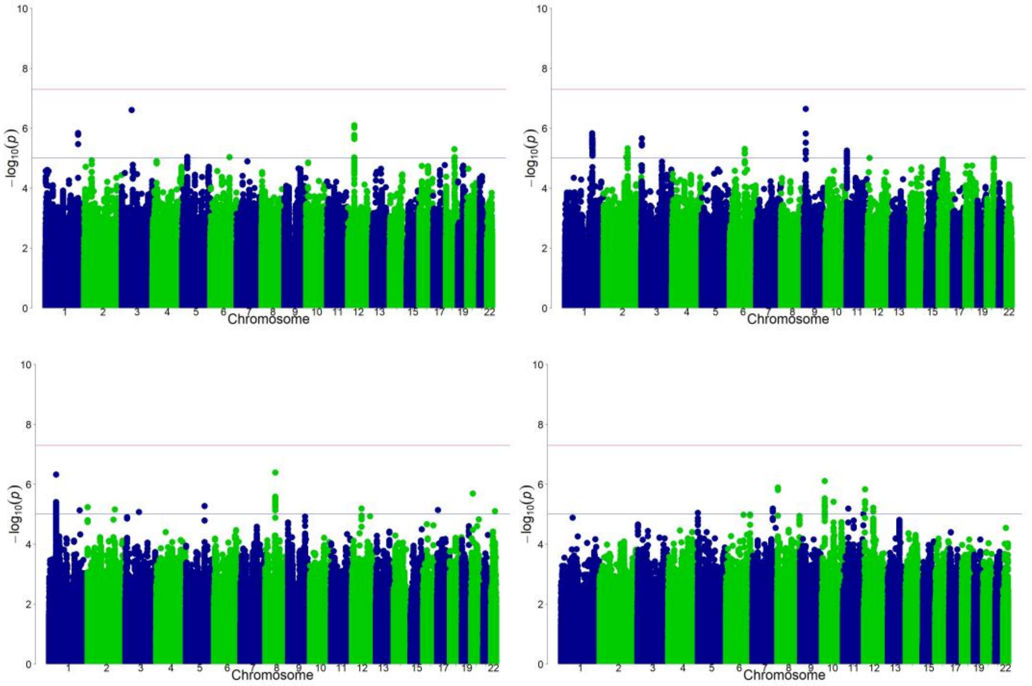

GWAS manhattan plot for headache

top left: BNT162b1 1st dose, top right: BNT162b1 2nd dose,

bottom left: mRNA-1273 1st dose, bottom right: mRNA-1273 2nd dose

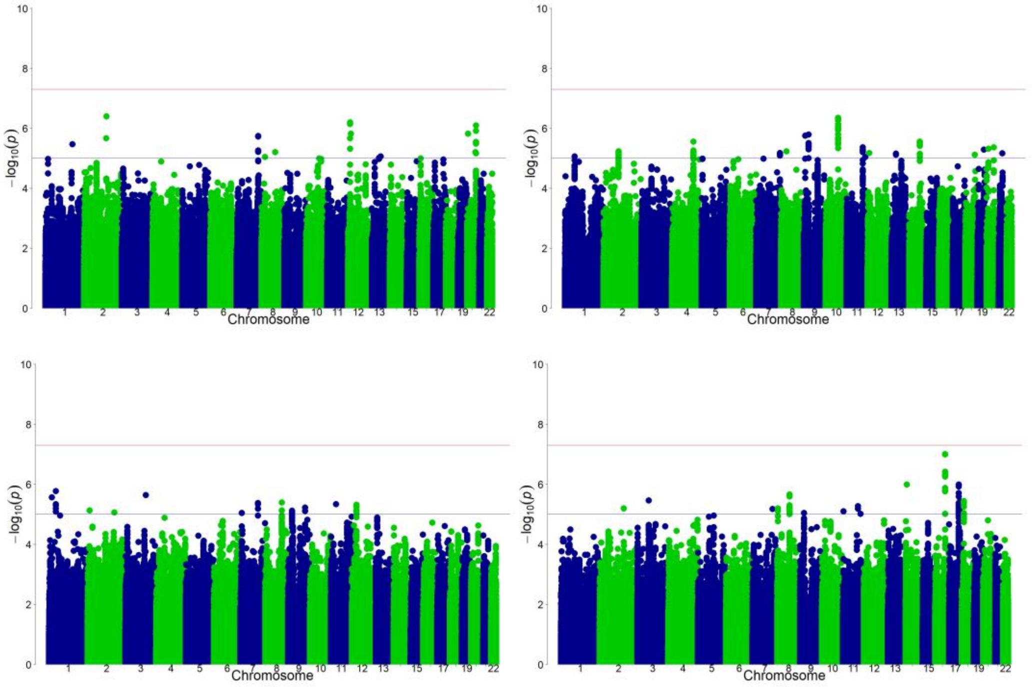

GWAS manhattan plot for sore throat

top left: BNT162b1 1st dose, top right: BNT162b1 2nd dose,

bottom left: mRNA-1273 1st dose, bottom right: mRNA-1273 2nd dose

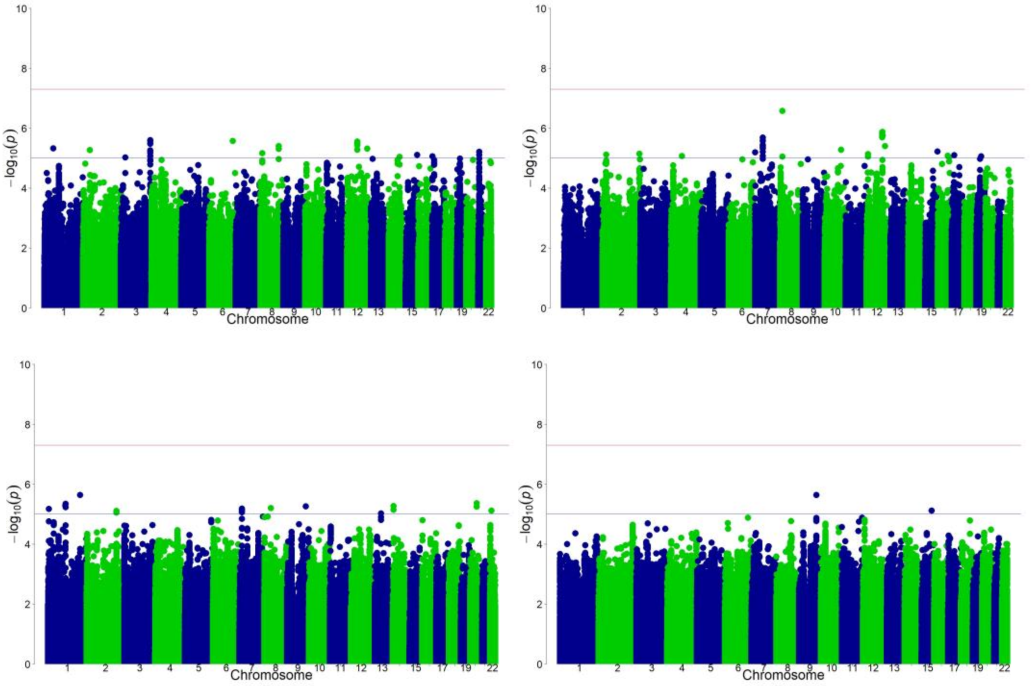

GWAS manhattan plot for mouth and throat discomfort

top left: BNT162b1 1st dose, top right: BNT162b1 2nd dose,

bottom left: mRNA-1273 1st dose, bottom right: mRNA-1273 2nd dose

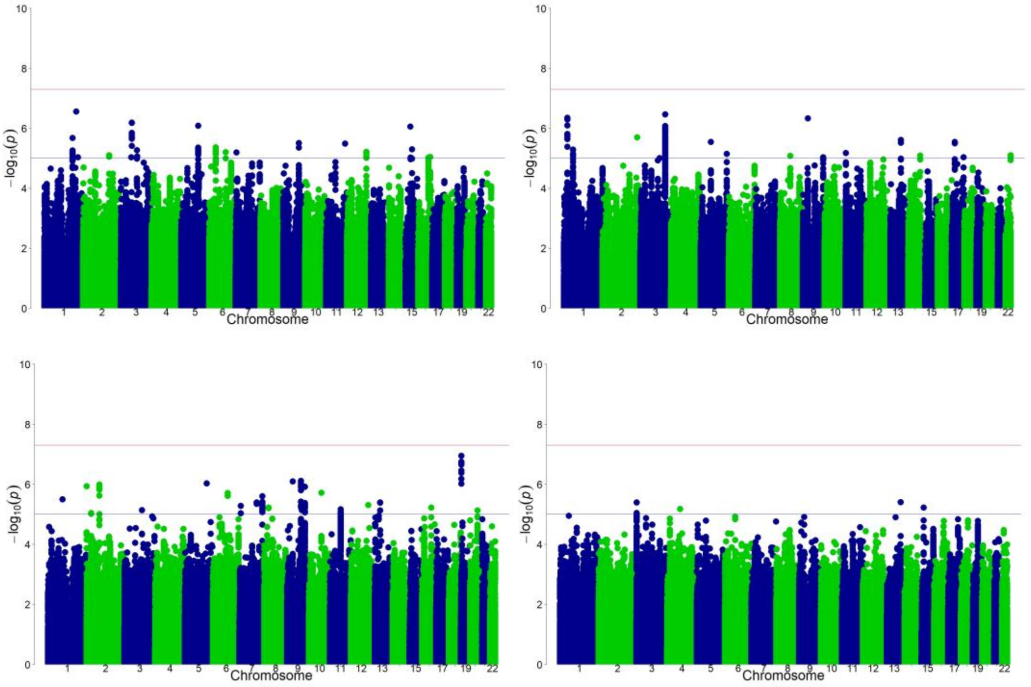

GWAS manhattan plot for back pain

top left: BNT162b1 1st dose, top right: BNT162b1 2nd dose,

bottom left: mRNA-1273 1st dose, bottom right: mRNA-1273 2nd dose

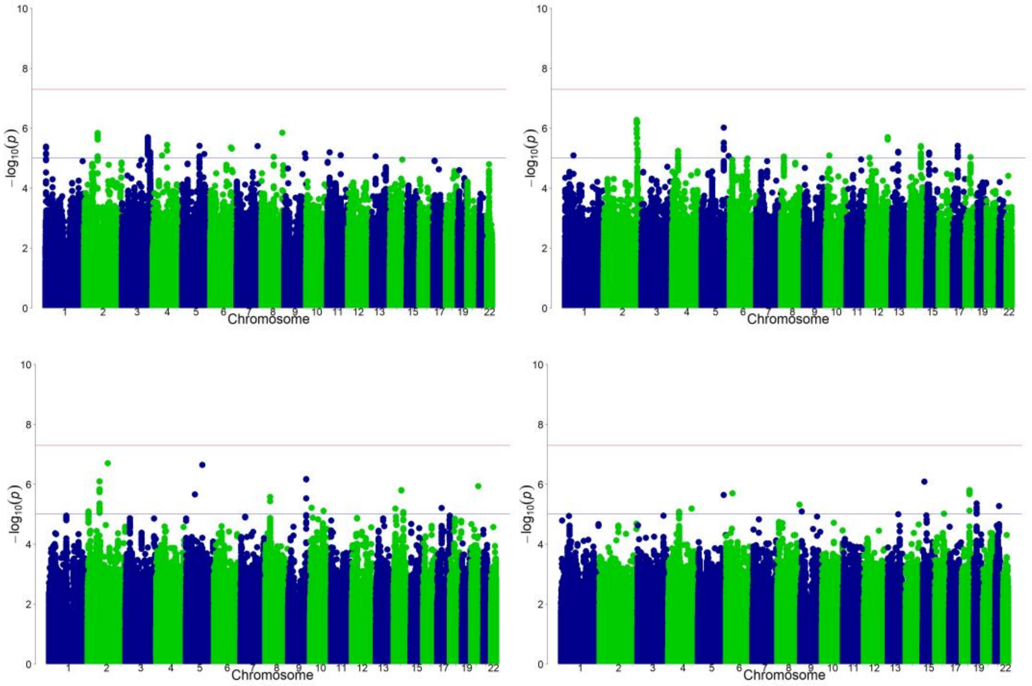

GWAS manhattan plot for swelling of lymph nodes (especially neck, armpit, inguinal part)

top left: BNT162b1 1st dose, top right: BNT162b1 2nd dose,

bottom left: mRNA-1273 1st dose, bottom right: mRNA-1273 2nd dose

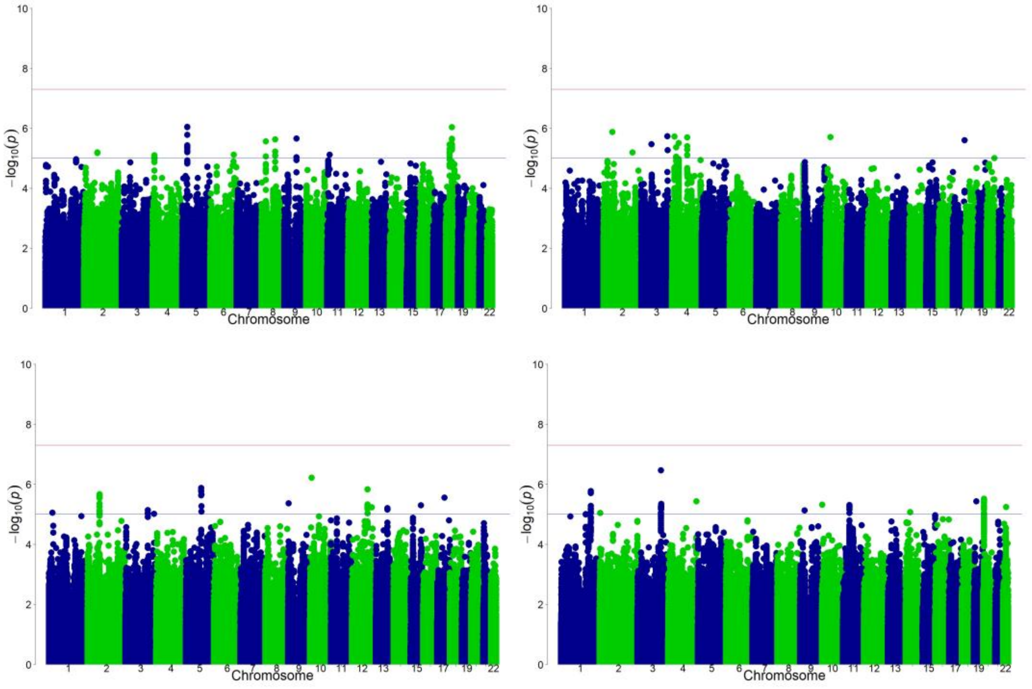

GWAS manhattan plot for pain of lymph nodes (especially neck, armpit, inguinal part)

top left: BNT162b1 1st dose, top right: BNT162b1 2nd dose,

bottom left: mRNA-1273 1st dose, bottom right: mRNA-1273 2nd dose

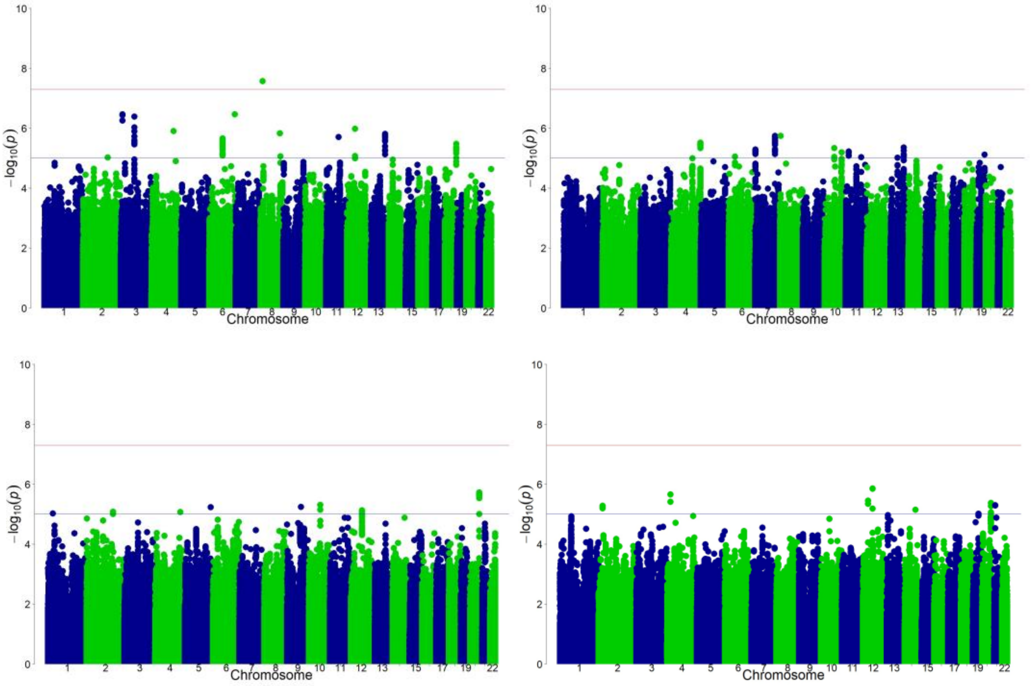

GWAS manhattan plot for chest pain

top left: BNT162b1 1st dose, top right: BNT162b1 2nd dose,

bottom left: mRNA-1273 1st dose, bottom right: mRNA-1273 2nd dose

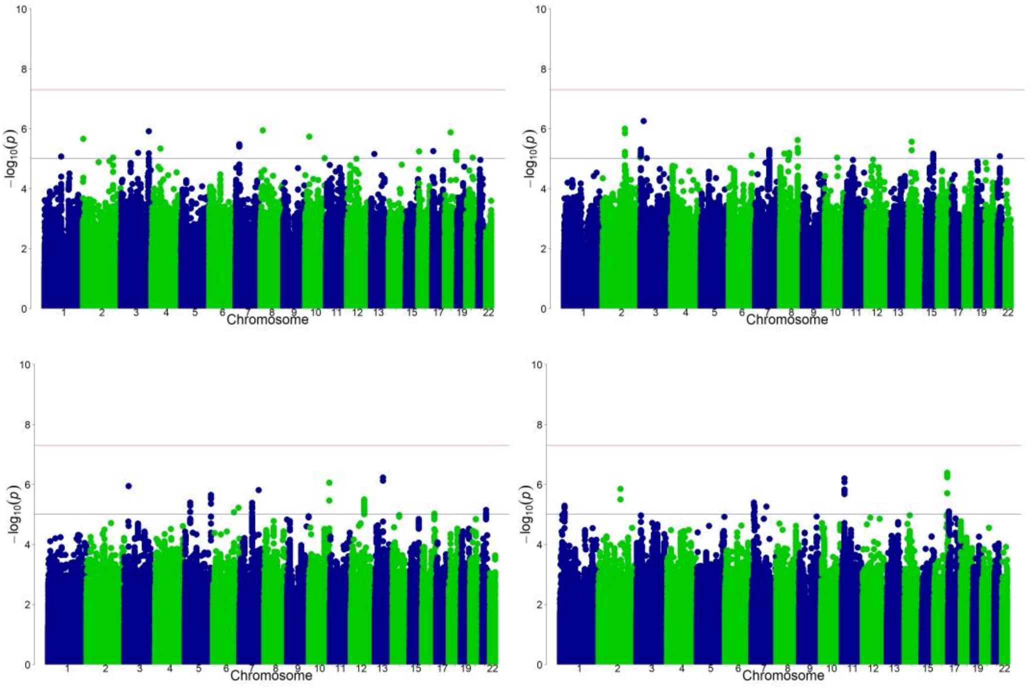

GWAS manhattan plot for stomach ache

top left: BNT162b1 1st dose, top right: BNT162b1 2nd dose,

bottom left: mRNA-1273 1st dose, bottom right: mRNA-1273 2nd dose

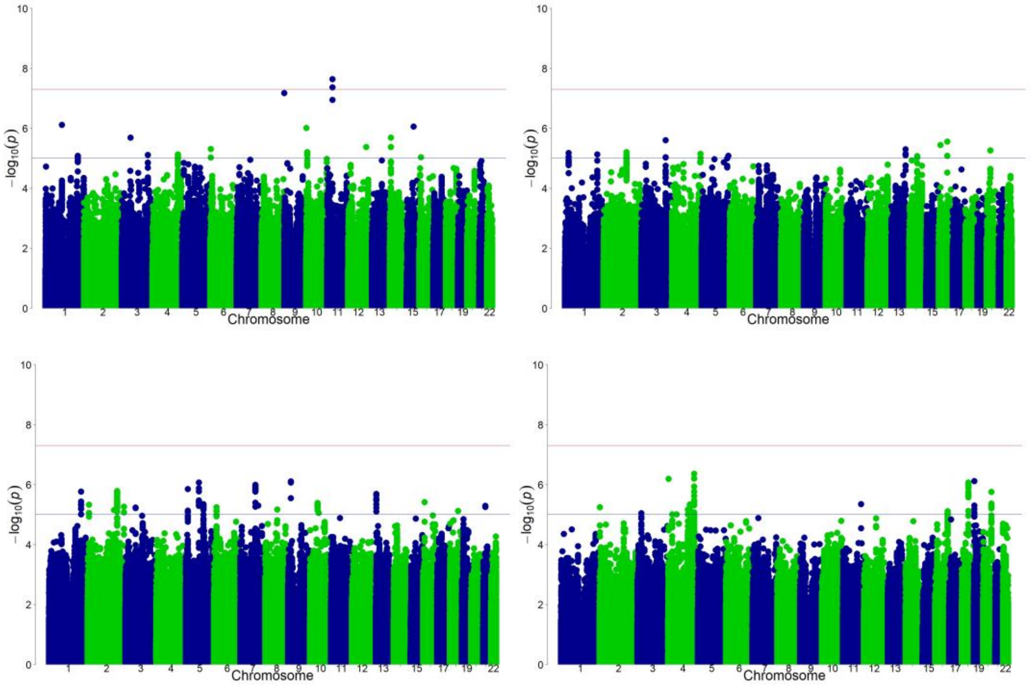

GWAS manhattan plot for abdominal discomfort

top left: BNT162b1 1st dose, top right: BNT162b1 2nd dose,

bottom left: mRNA-1273 1st dose, bottom right: mRNA-1273 2nd dose

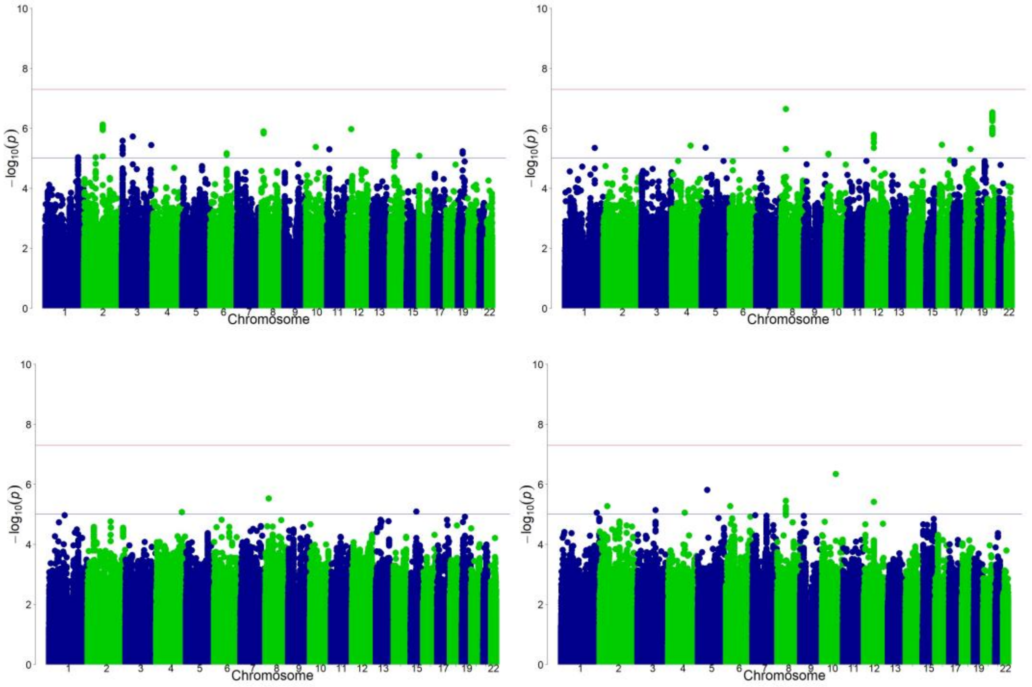

GWAS manhattan plot for eye pain

top left: BNT162b1 1st dose, top right: BNT162b1 2nd dose,

bottom left: mRNA-1273 1st dose, bottom right: mRNA-1273 2nd dose

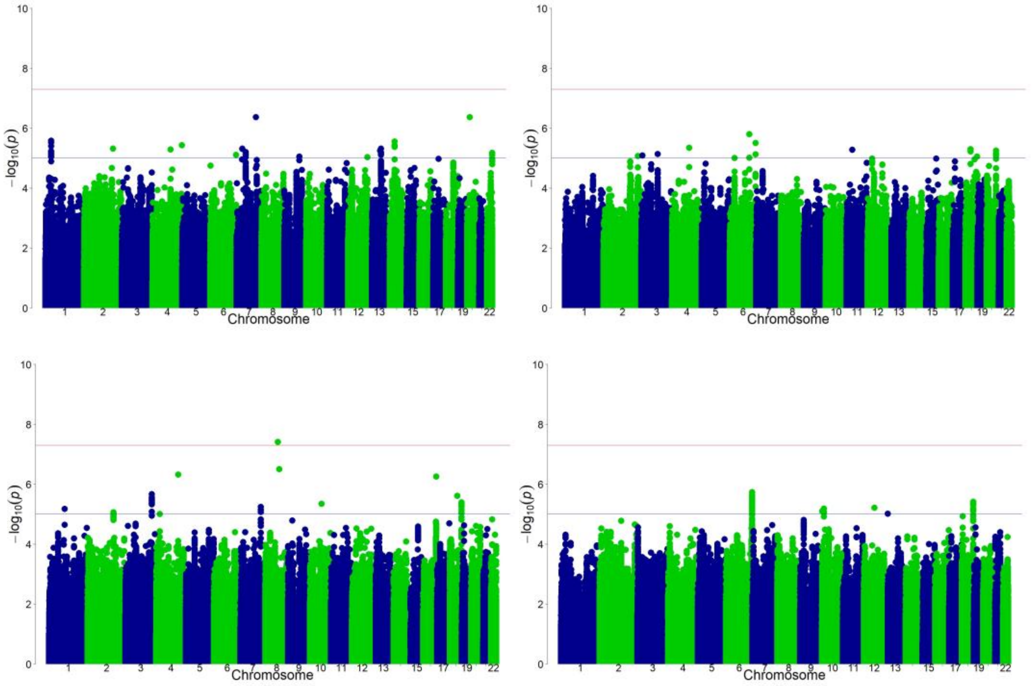

GWAS manhattan plot for joint pain

top left: BNT162b1 1st dose, top right: BNT162b1 2nd dose,

bottom left: mRNA-1273 1st dose, bottom right: mRNA-1273 2nd dose

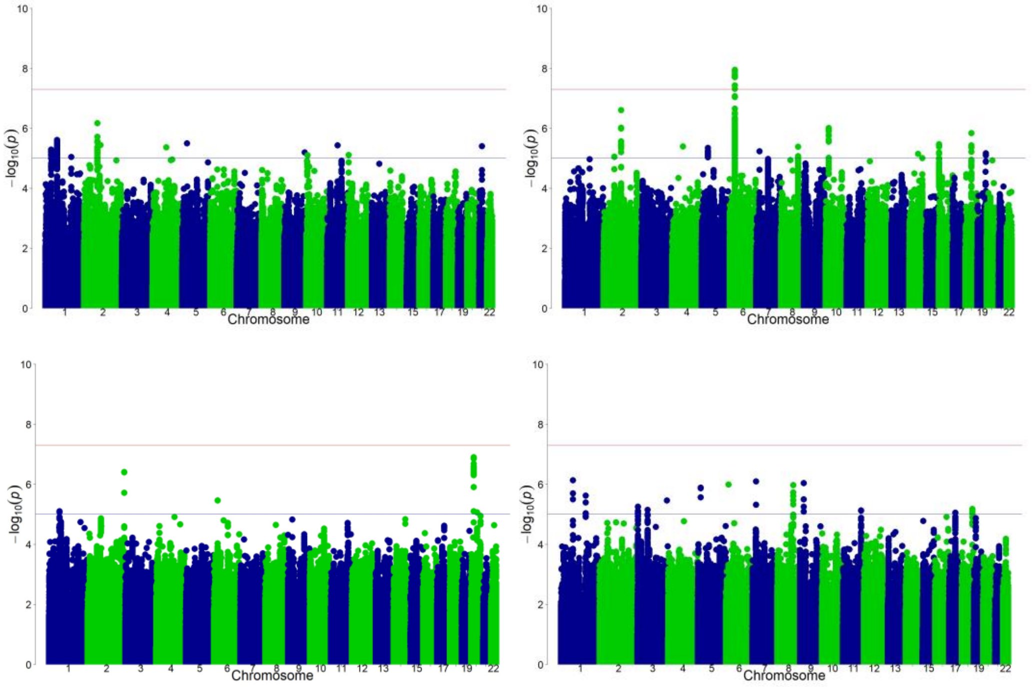

GWAS manhattan plot for muscle pain

top left: BNT162b1 1st dose, top right: BNT162b1 2nd dose,

bottom left: mRNA-1273 1st dose, bottom right: mRNA-1273 2nd dose

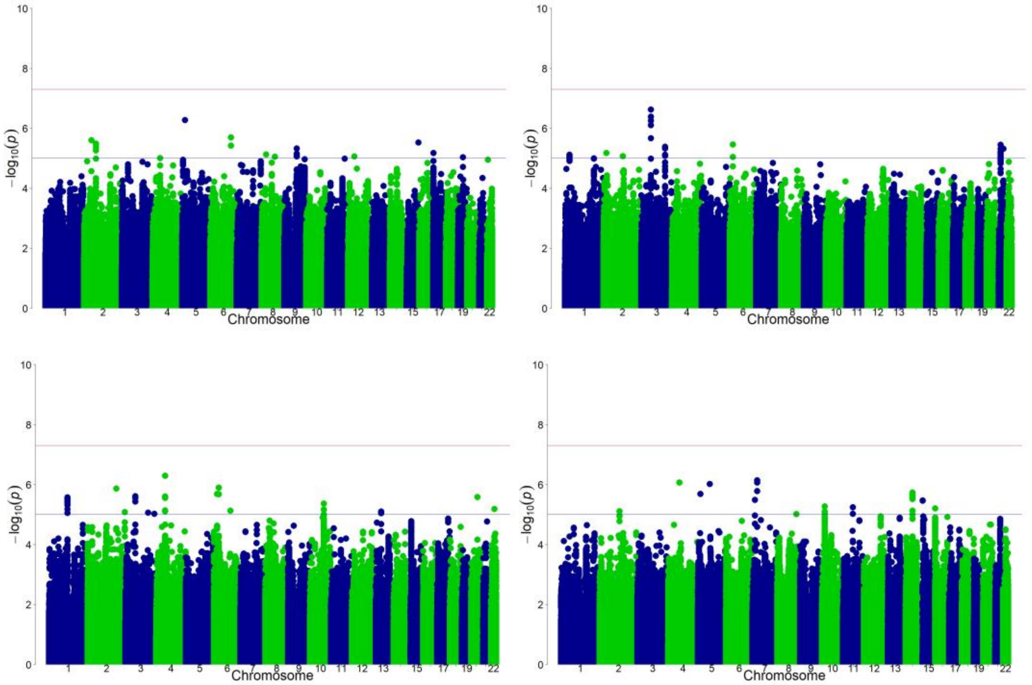

GWAS manhattan plot for musculoskeletal discomfort (such as muscle tension)

top left: BNT162b1 1st dose, top right: BNT162b1 2nd dose,

bottom left: mRNA-1273 1st dose, bottom right: mRNA-1273 2nd dose

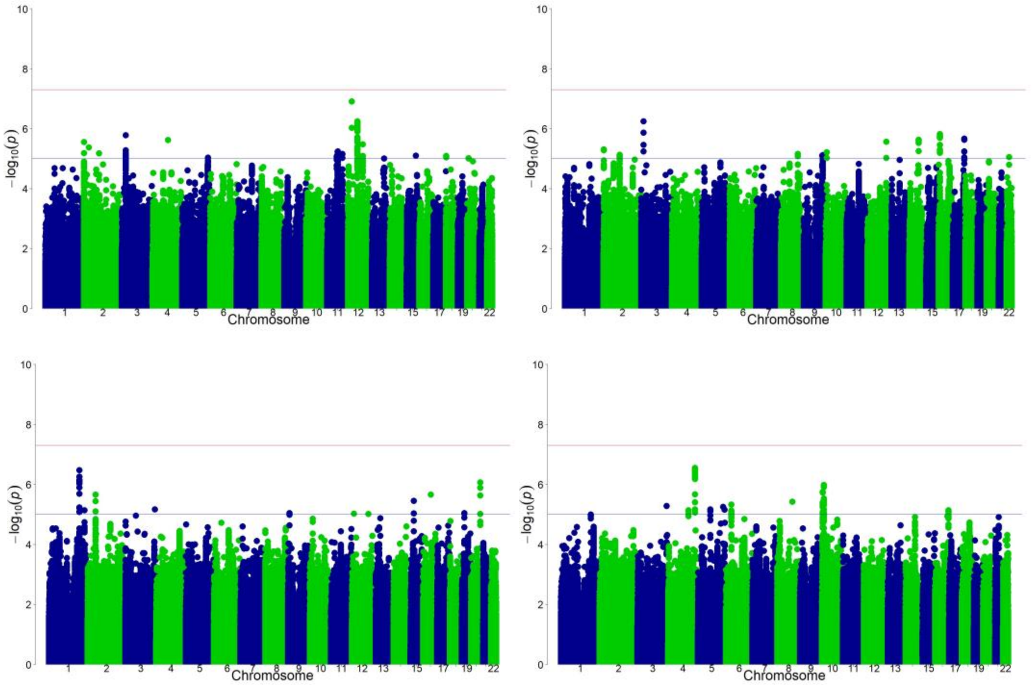

GWAS manhattan plot for limb pain

top left: BNT162b1 1st dose, top right: BNT162b1 2nd dose,

bottom left: mRNA-1273 1st dose, bottom right: mRNA-1273 2nd dose

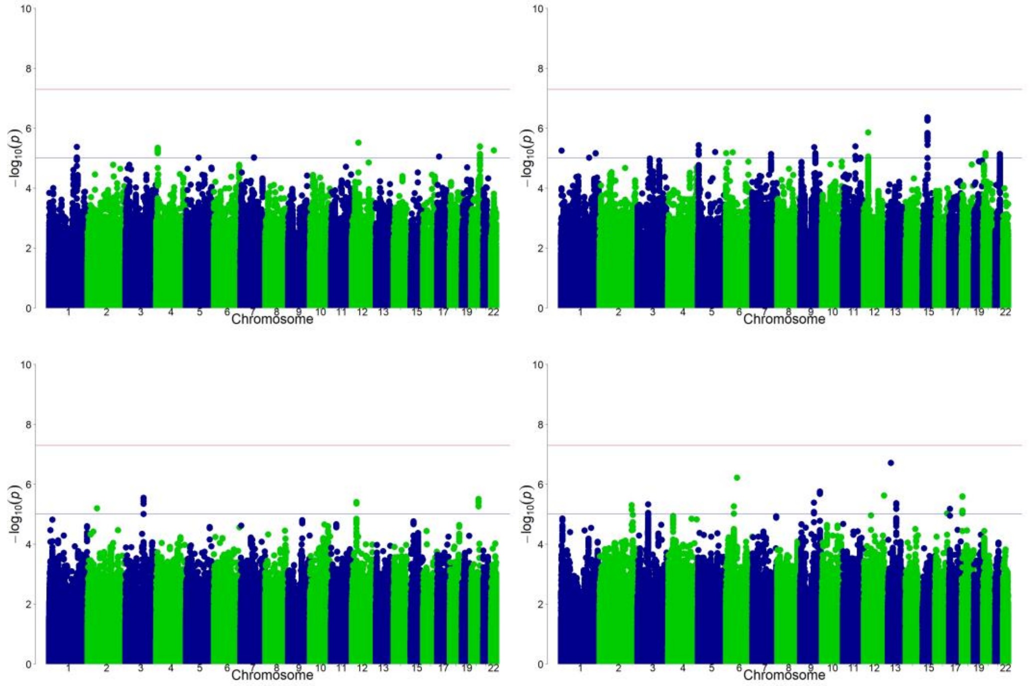

GWAS manhattan plot for limb discomfort

top left: BNT162b1 1st dose, top right: BNT162b1 2nd dose,

bottom left: mRNA-1273 1st dose, bottom right: mRNA-1273 2nd dose

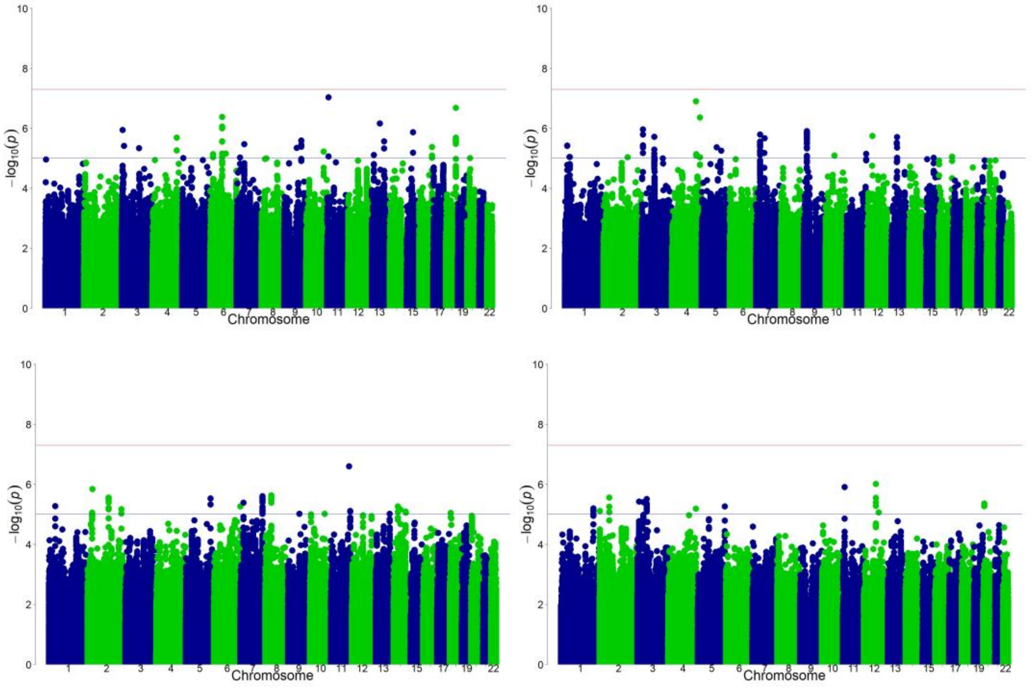

GWAS manhattan plot for snot

top left: BNT162b1 1st dose, top right: BNT162b1 2nd dose,

bottom left: mRNA-1273 1st dose, bottom right: mRNA-1273 2nd dose

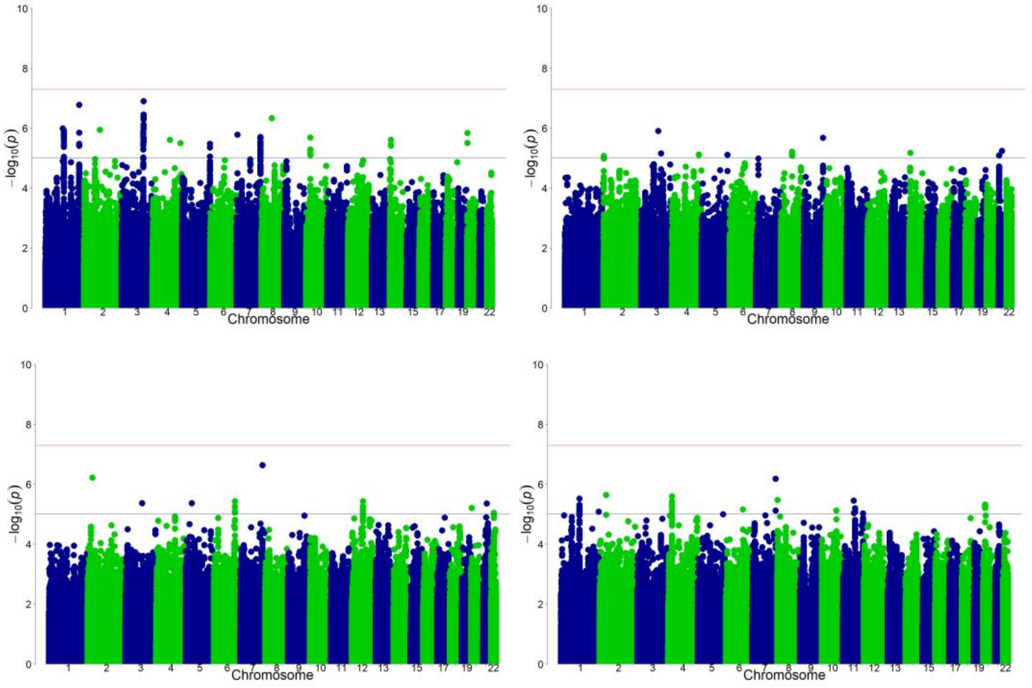

GWAS manhattan plot for stuffy nose

top left: BNT162b1 1st dose, top right: BNT162b1 2nd dose,

bottom left: mRNA-1273 1st dose, bottom right: mRNA-1273 2nd dose

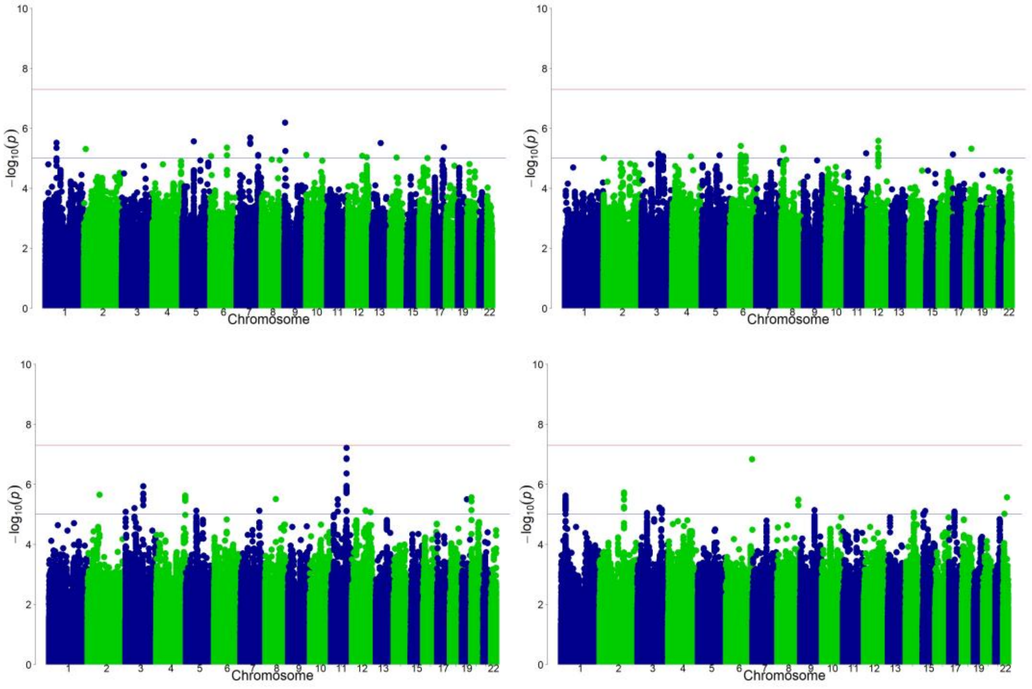

GWAS manhattan plot for nausea

top left: BNT162b1 1st dose, top right: BNT162b1 2nd dose,

bottom left: mRNA-1273 1st dose, bottom right: mRNA-1273 2nd dose

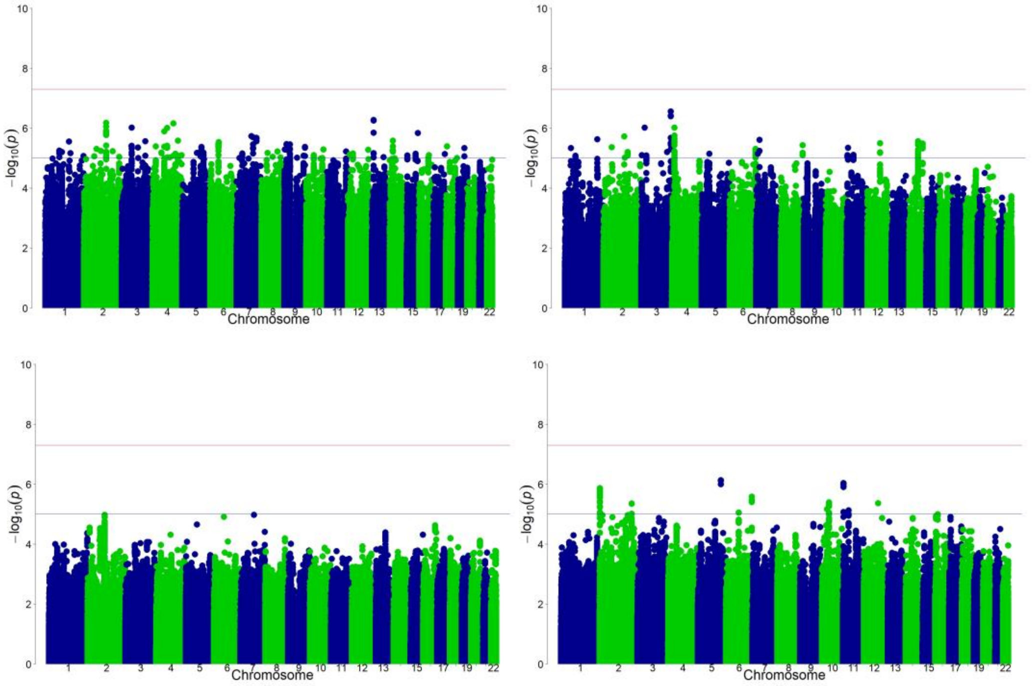

GWAS manhattan plot for vomiting

top left: BNT162b1 1st dose, top right: BNT162b1 2nd dose,

bottom left: mRNA-1273 1st dose, bottom right: mRNA-1273 2nd dose

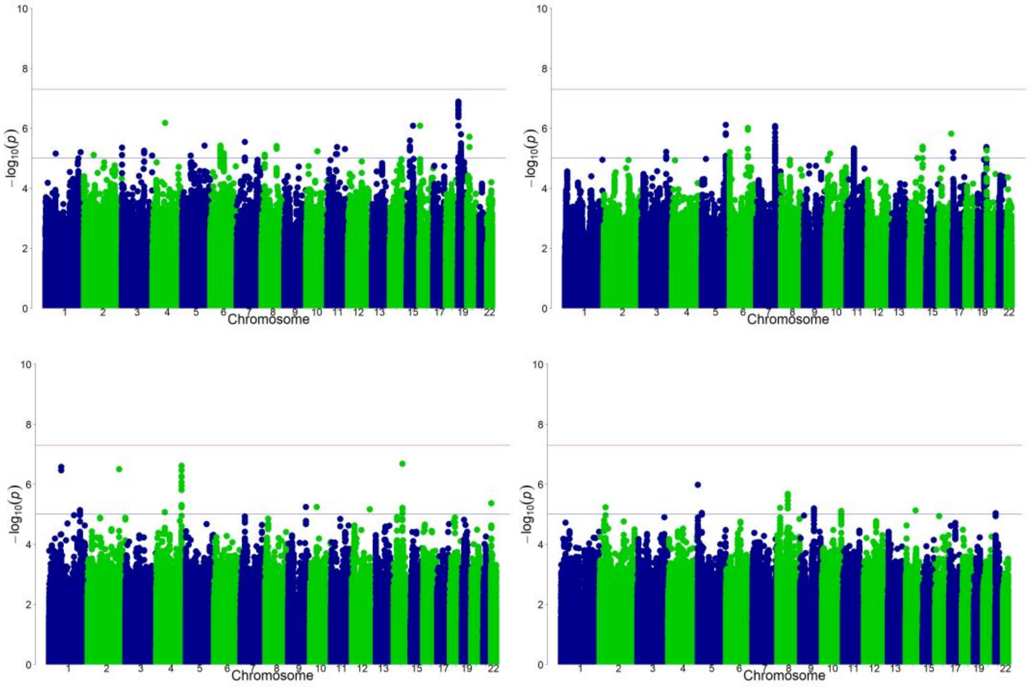

GWAS manhattan plot for loss of appetite

top left: BNT162b1 1st dose, top right: BNT162b1 2nd dose,

bottom left: mRNA-1273 1st dose, bottom right: mRNA-1273 2nd dose

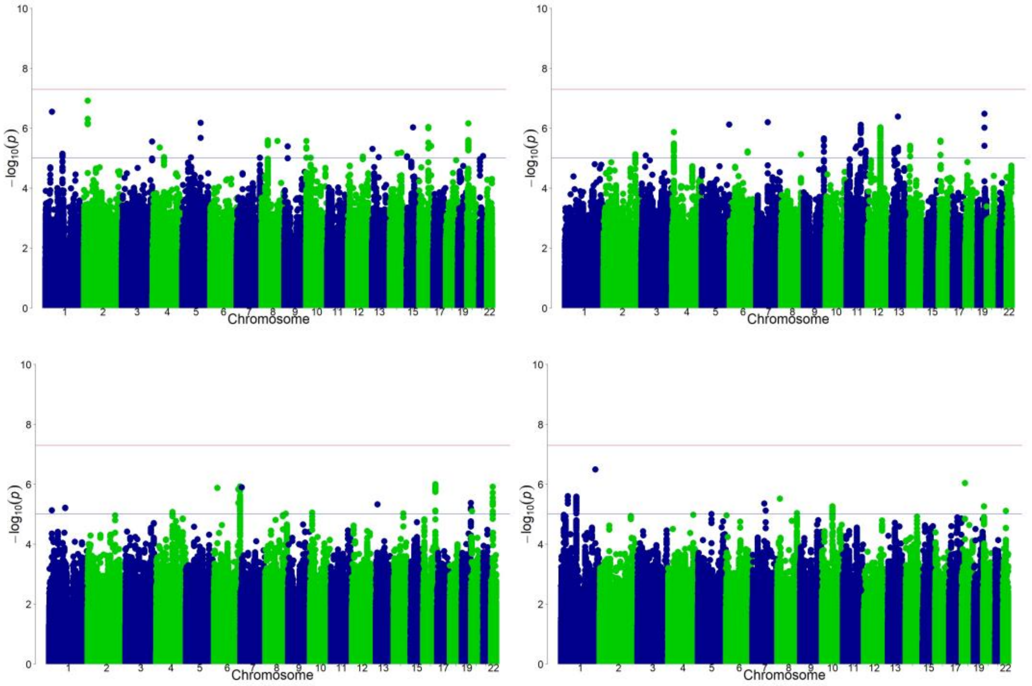

GWAS manhattan plot for diarrhea

top left: BNT162b1 1st dose, top right: BNT162b1 2nd dose,

bottom left: mRNA-1273 1st dose, bottom right: mRNA-1273 2nd dose

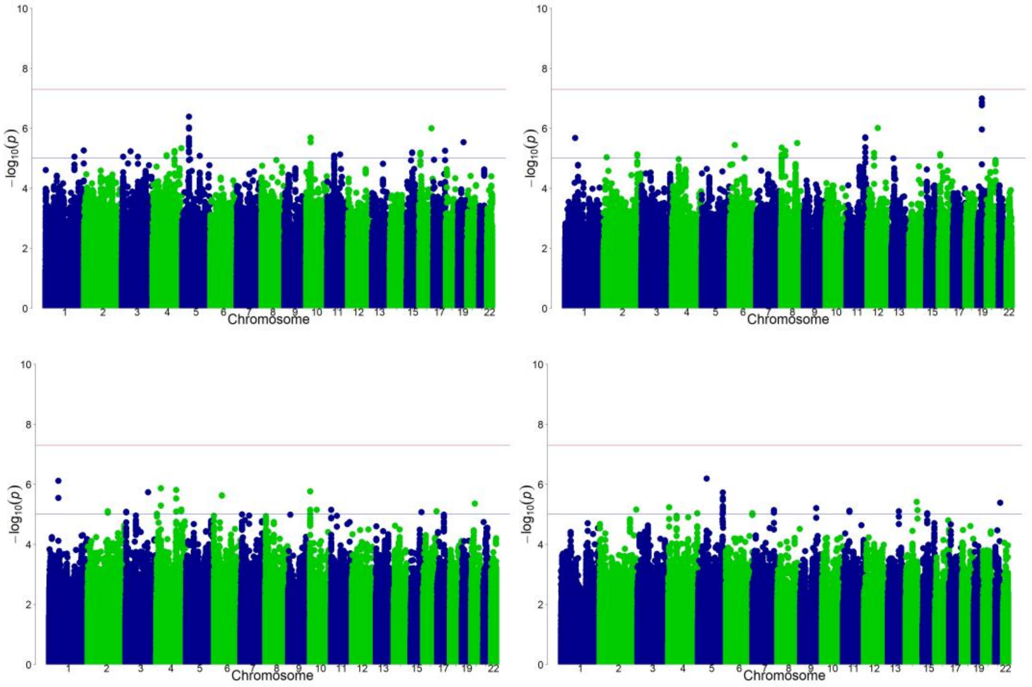

GWAS manhattan plot for loose stool

top left: BNT162b1 1st dose, top right: BNT162b1 2nd dose,

bottom left: mRNA-1273 1st dose, bottom right: mRNA-1273 2nd dose

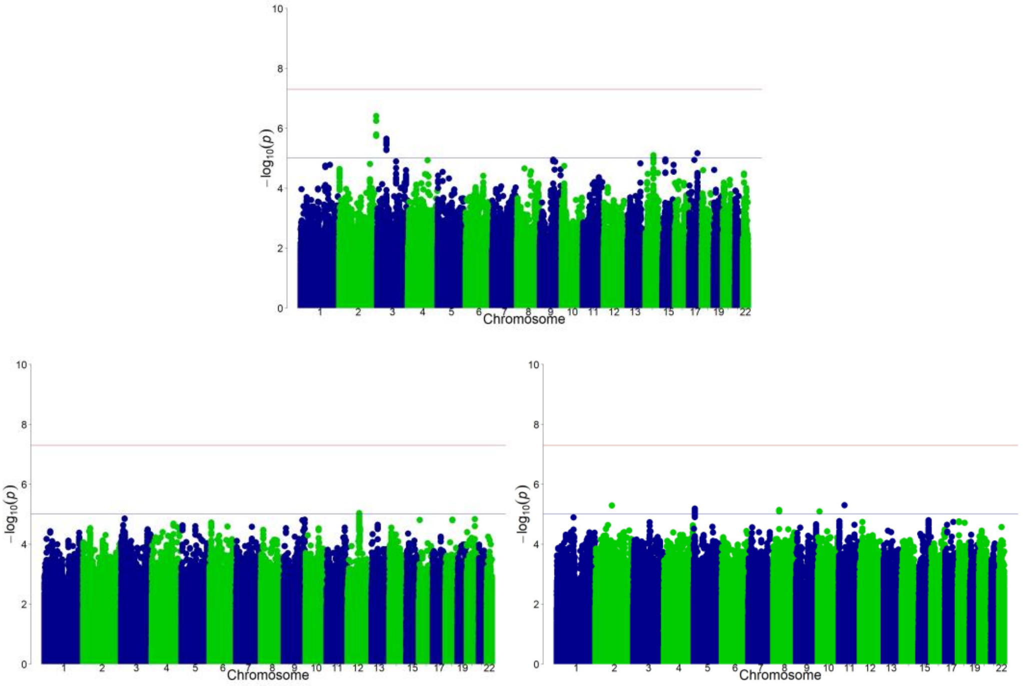

GWAS manhattan plot for constipation

top: BNT162b1 2nd dose,

bottom left: mRNA-1273 1st dose, bottom right: mRNA-1273 2nd dose

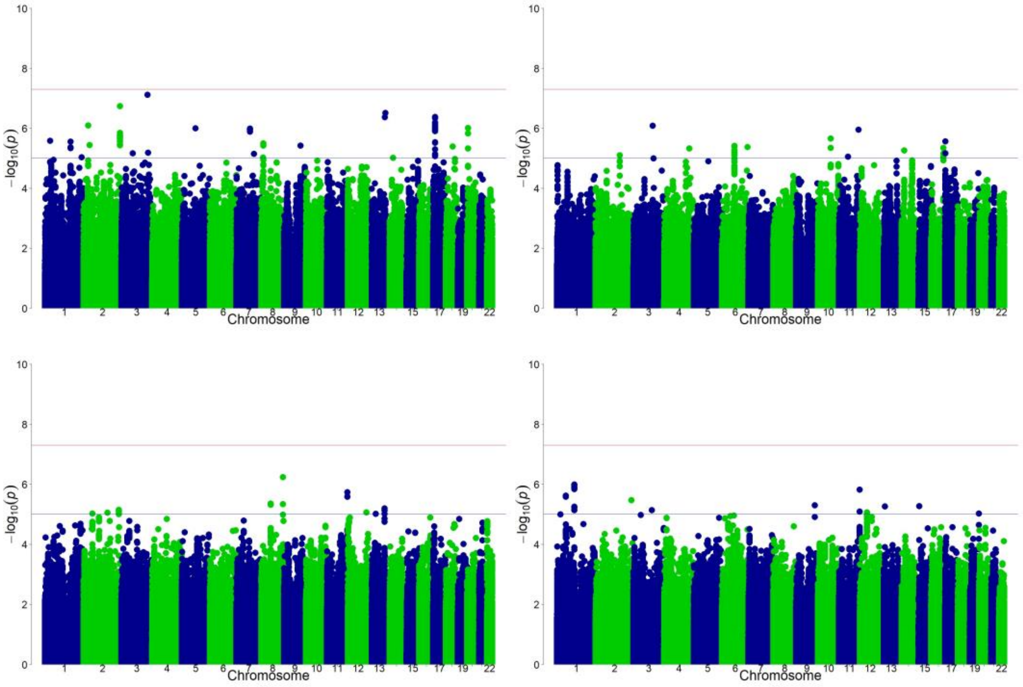

GWAS manhattan plot for cough

top left: BNT162b1 1st dose, top right: BNT162b1 2nd dose,

bottom left: mRNA-1273 1st dose, bottom right: mRNA-1273 2nd dose

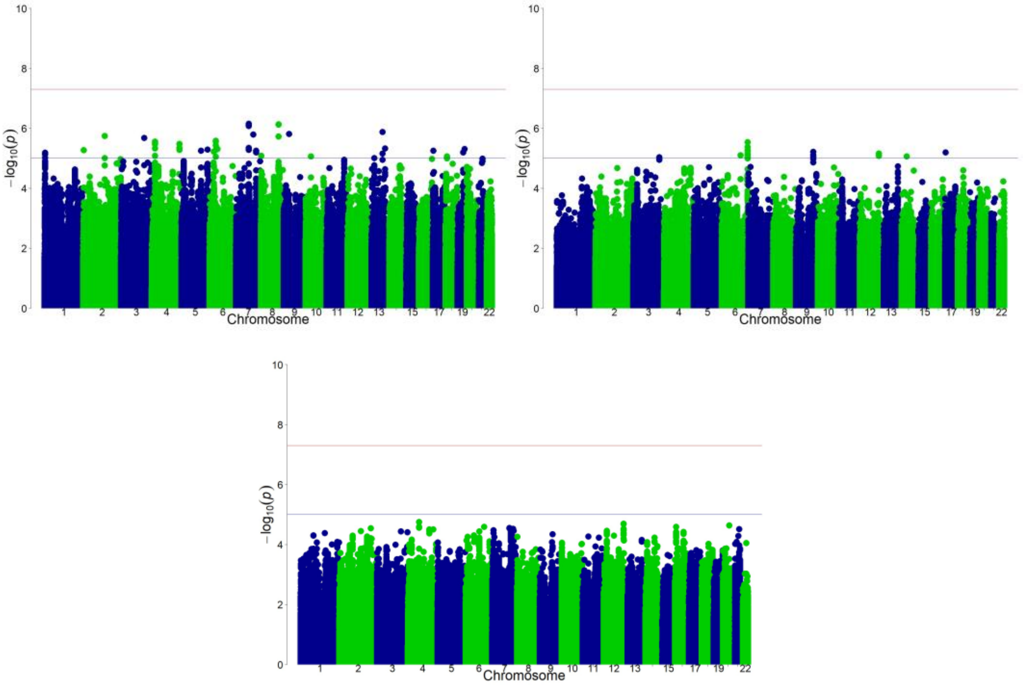

GWAS manhattan plot for sneeze

top left: BNT162b1 1st dose, top right: BNT162b1 2nd dose,

bottom: mRNA-1273 2nd dose

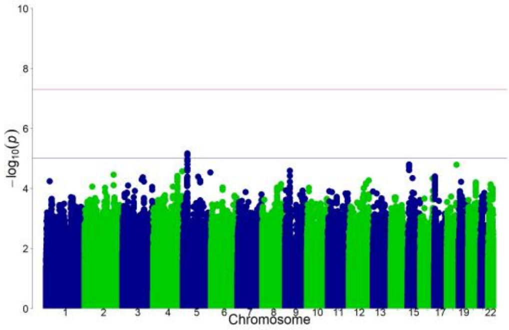

GWAS manhattan plot for dyspnea at mRNA-1273 2nd dose

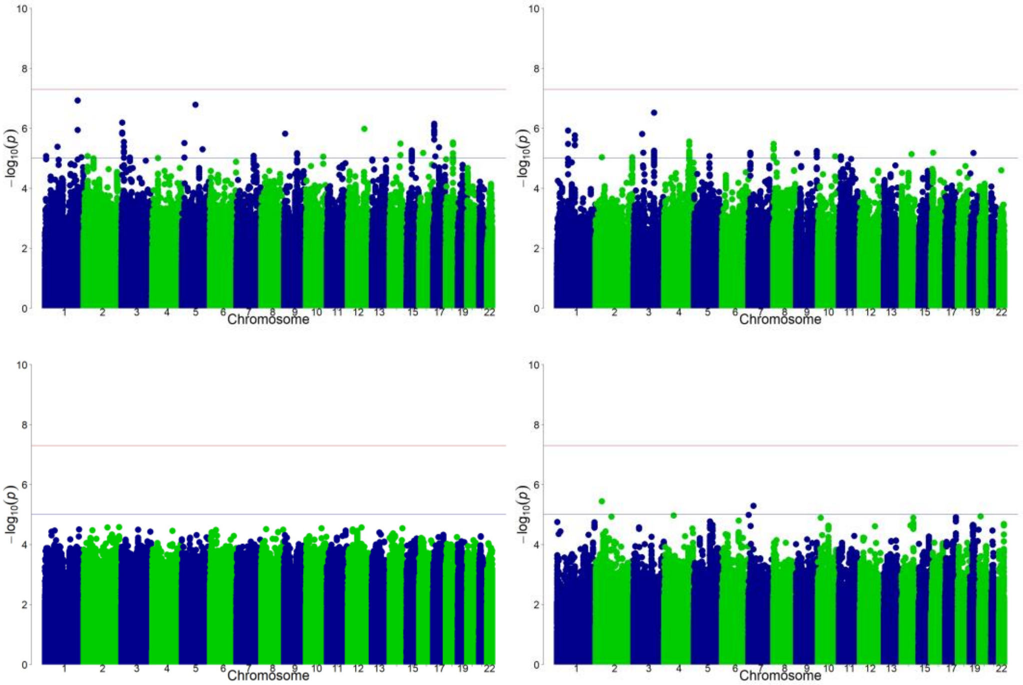

GWAS manhattan plot for muscle weakness

top left: BNT162b1 1st dose, top right: BNT162b1 2nd dose,

bottom left: mRNA-1273 1st dose, bottom right: mRNA-1273 2nd dose

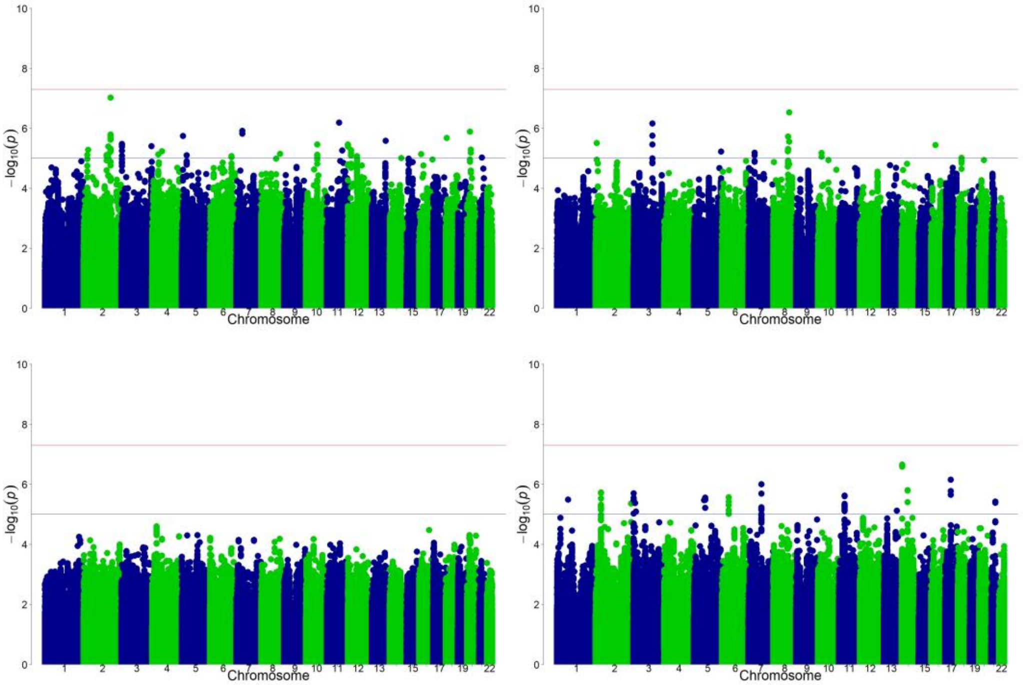

GWAS manhattan plot for stomatitis

top left: BNT162b1 1st dose, top right: BNT162b1 2nd dose,

bottom left: mRNA-1273 1st dose, bottom right: mRNA-1273 2nd dose

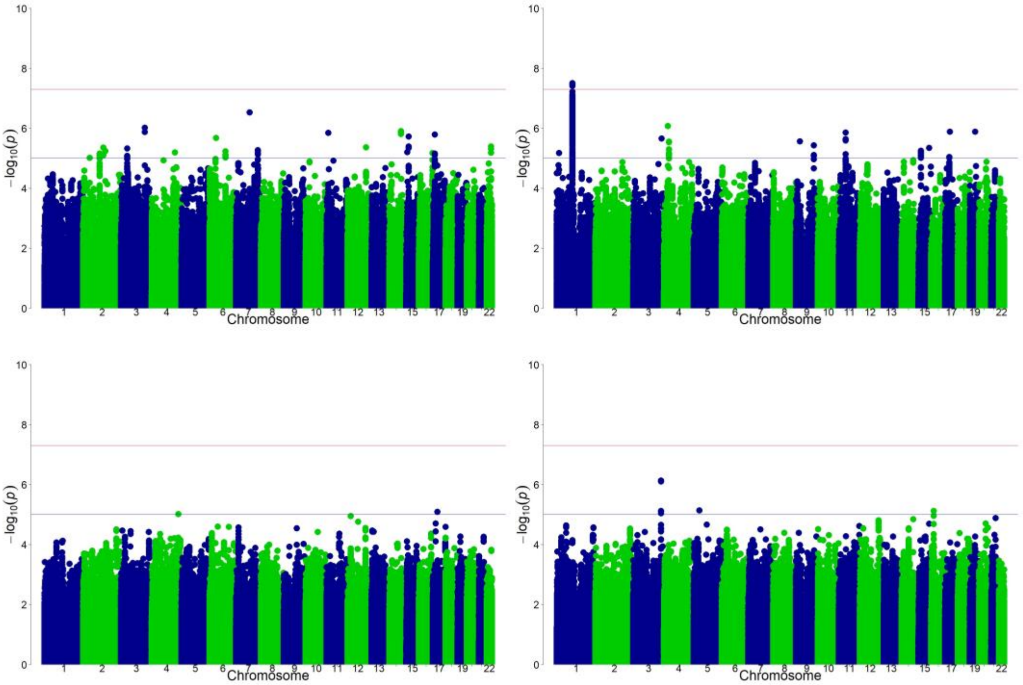

GWAS manhattan plot for urticaria (rash that disappears within a few hours)

top left: BNT162b1 1st dose, top right: BNT162b1 2nd dose,

bottom left: mRNA-1273 1st dose, bottom right: mRNA-1273 2nd dose

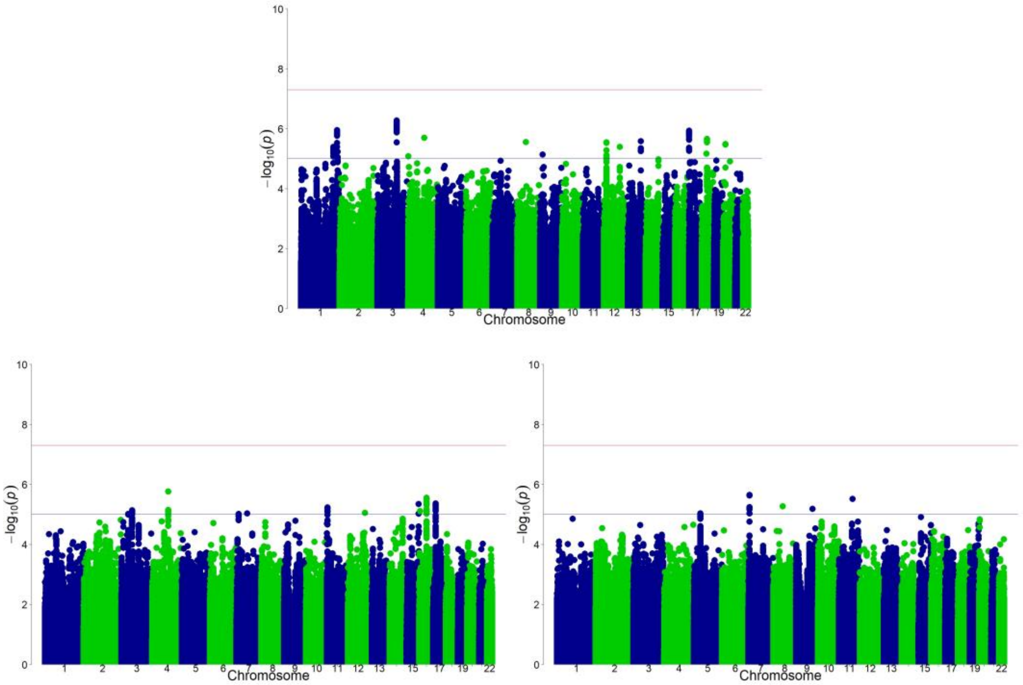

GWAS manhattan plot for eczema (long-term rash)

top: BNT162b1 2nd dose,

bottom left: mRNA-1273 1st dose, bottom right: mRNA-1273 2nd dose

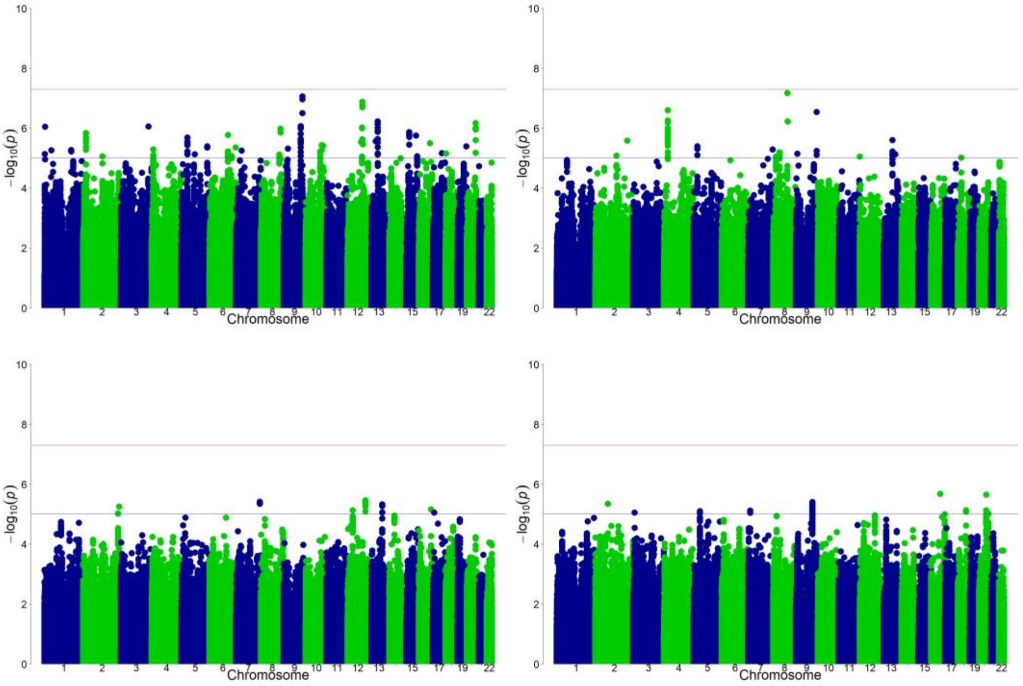

GWAS manhattan plot for blunt feeling

top left: BNT162b1 1st dose, top right: BNT162b1 2nd dose,

bottom left: mRNA-1273 1st dose, bottom right: mRNA-1273 2nd dose

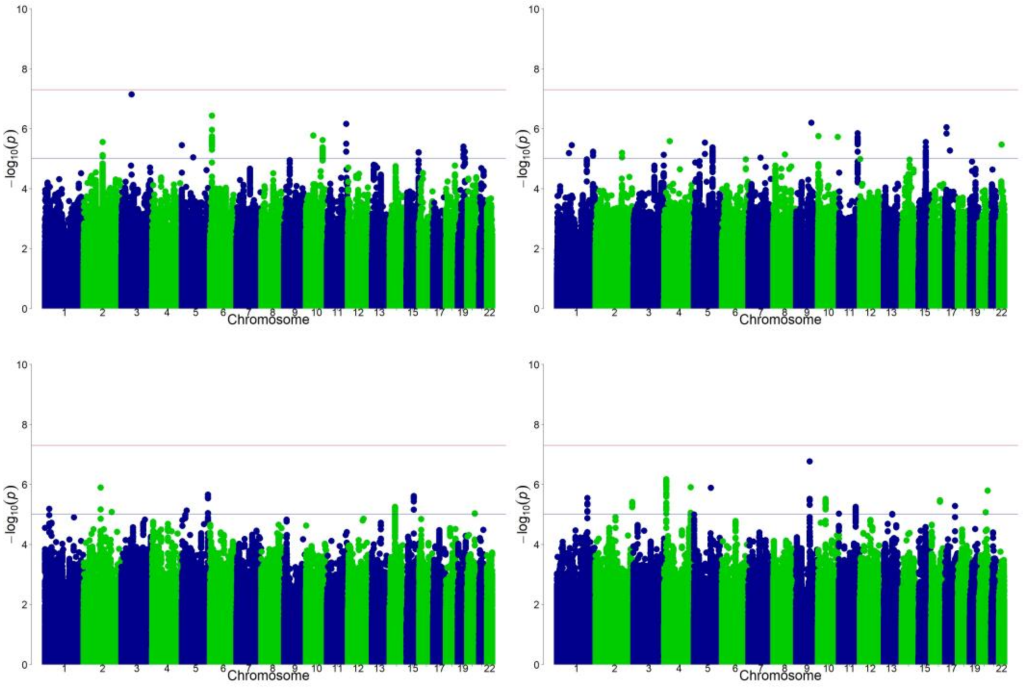

GWAS manhattan plot for sleepy in the daytime

top left: BNT162b1 1st dose, top right: BNT162b1 2nd dose,

bottom left: mRNA-1273 1st dose, bottom right: mRNA-1273 2nd dose

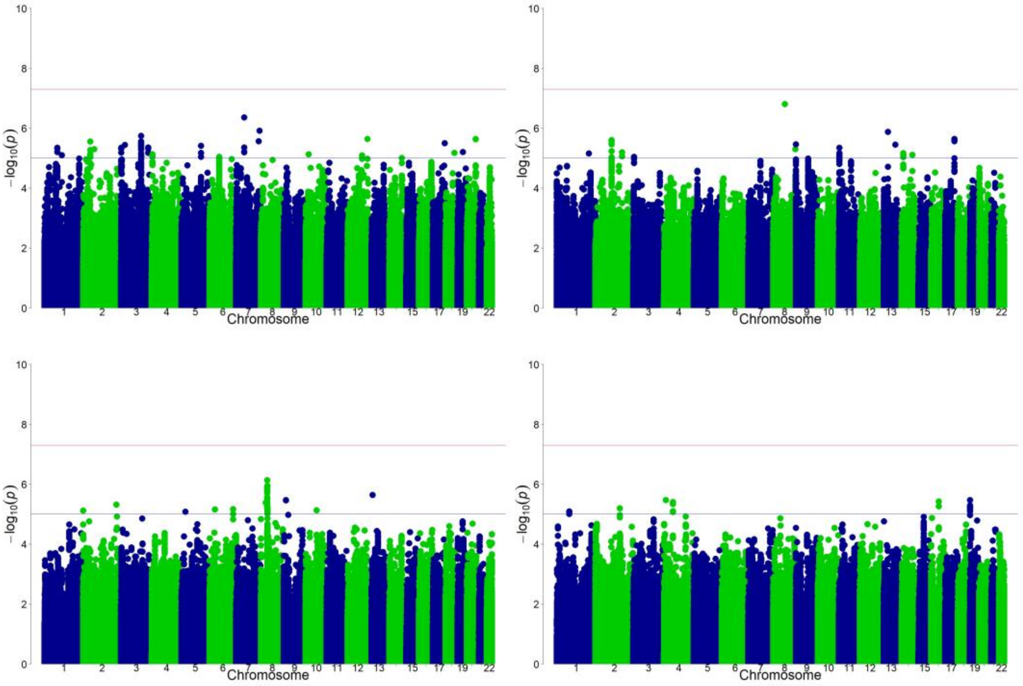

GWAS manhattan plot for insomnia

top left: BNT162b1 1st dose, top right: BNT162b1 2nd dose,

bottom left: mRNA-1273 1st dose, bottom right: mRNA-1273 2nd dose

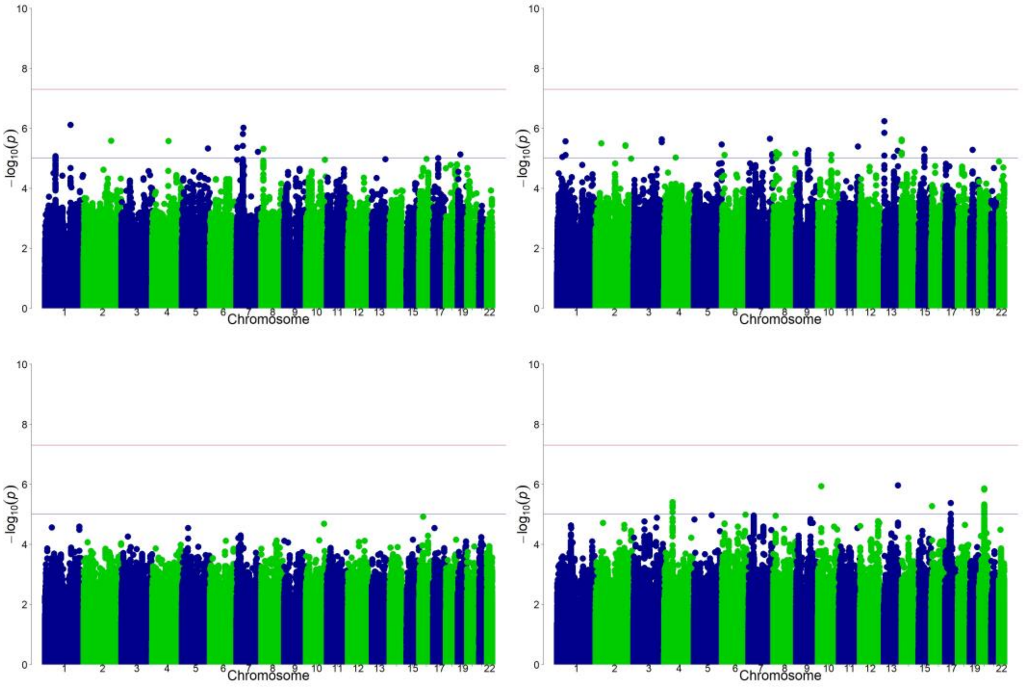

GWAS manhattan plot for hyperhidrosis

top left: BNT162b1 1st dose, top right: BNT162b1 2nd dose,

bottom left: mRNA-1273 1st dose, bottom right: mRNA-1273 2nd dose

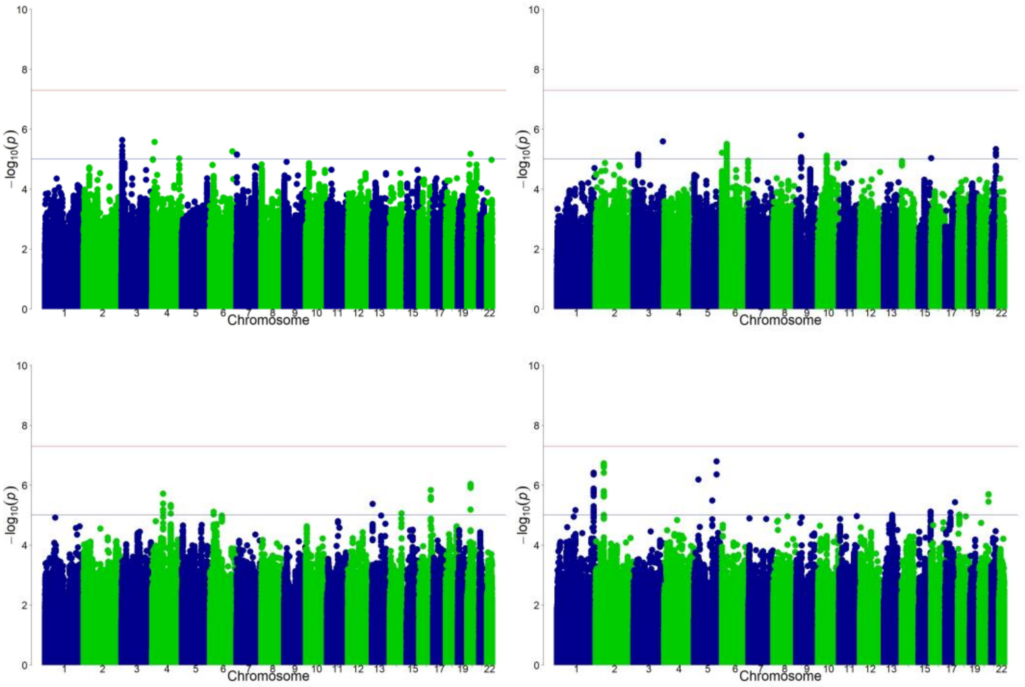

GWAS manhattan plot for there is nothing that applies in this (systemic reaction)

top left: BNT162b1 1st dose, top right: BNT162b1 2nd dose,

bottom left: mRNA-1273 1st dose, bottom right: mRNA-1273 2nd dose

Supplementary Figure 1. GWAS manhattan plots of meta-analysis for COVID-19 vaccine adverse event

Due to the absence of cases in either population, we were unable to perform GWAS for the following conditions, we were unable to perform GWAS for following conditions.

constipation at BNT162b1 1st dose

dyspnea at BNT162b1 1st and 2nd dose and mRNA-1273 1st dose

eczema (long-term rash) at BNT162b1 1st dose

sneeze at mRNA-1273 1st dose

HLA-DQA1*03:01 at 1st dose of BNT162b1 vaccine

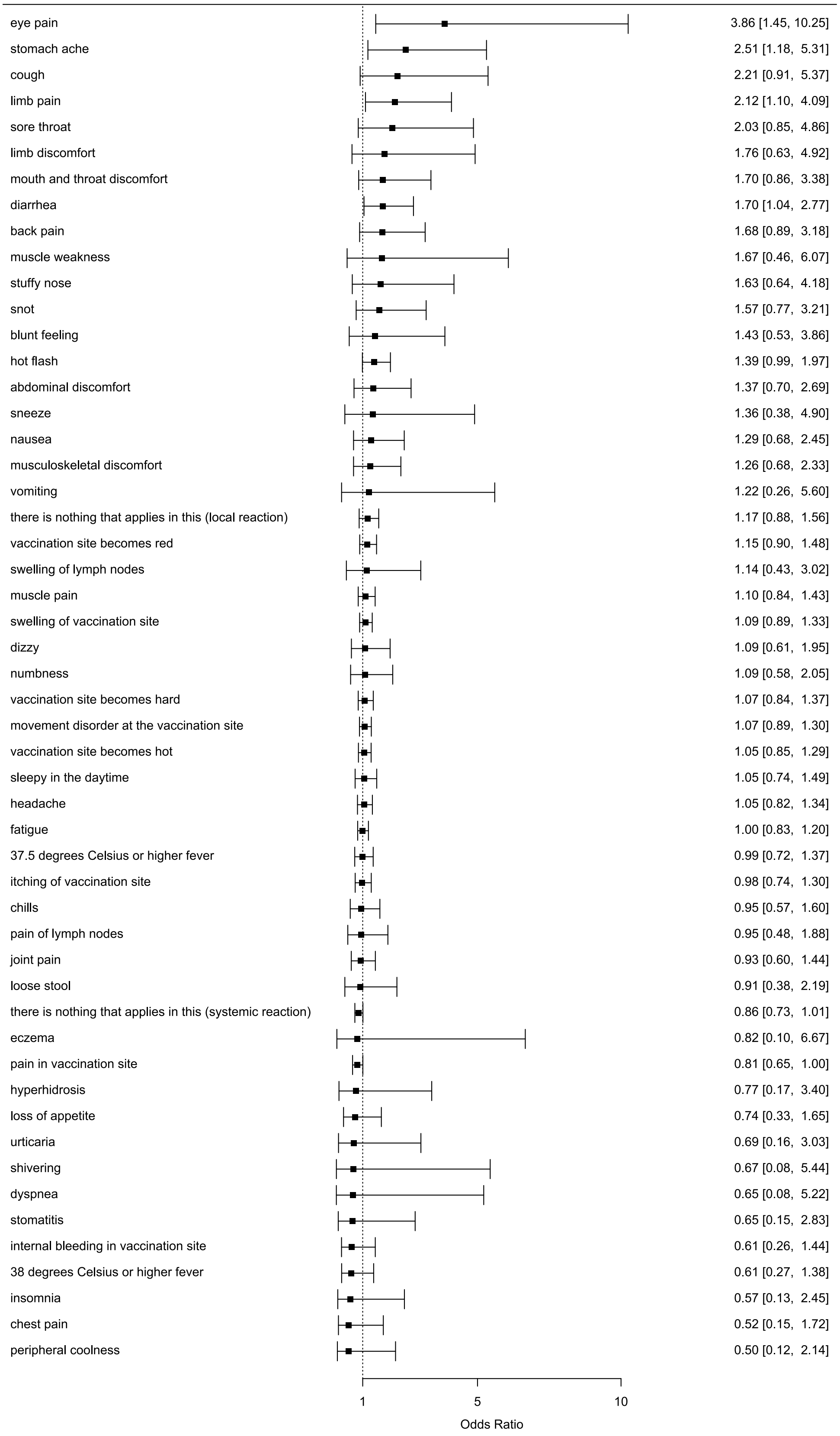

HLA-DQA1*03:01 at 2nd dose of BNT162b1 vaccine

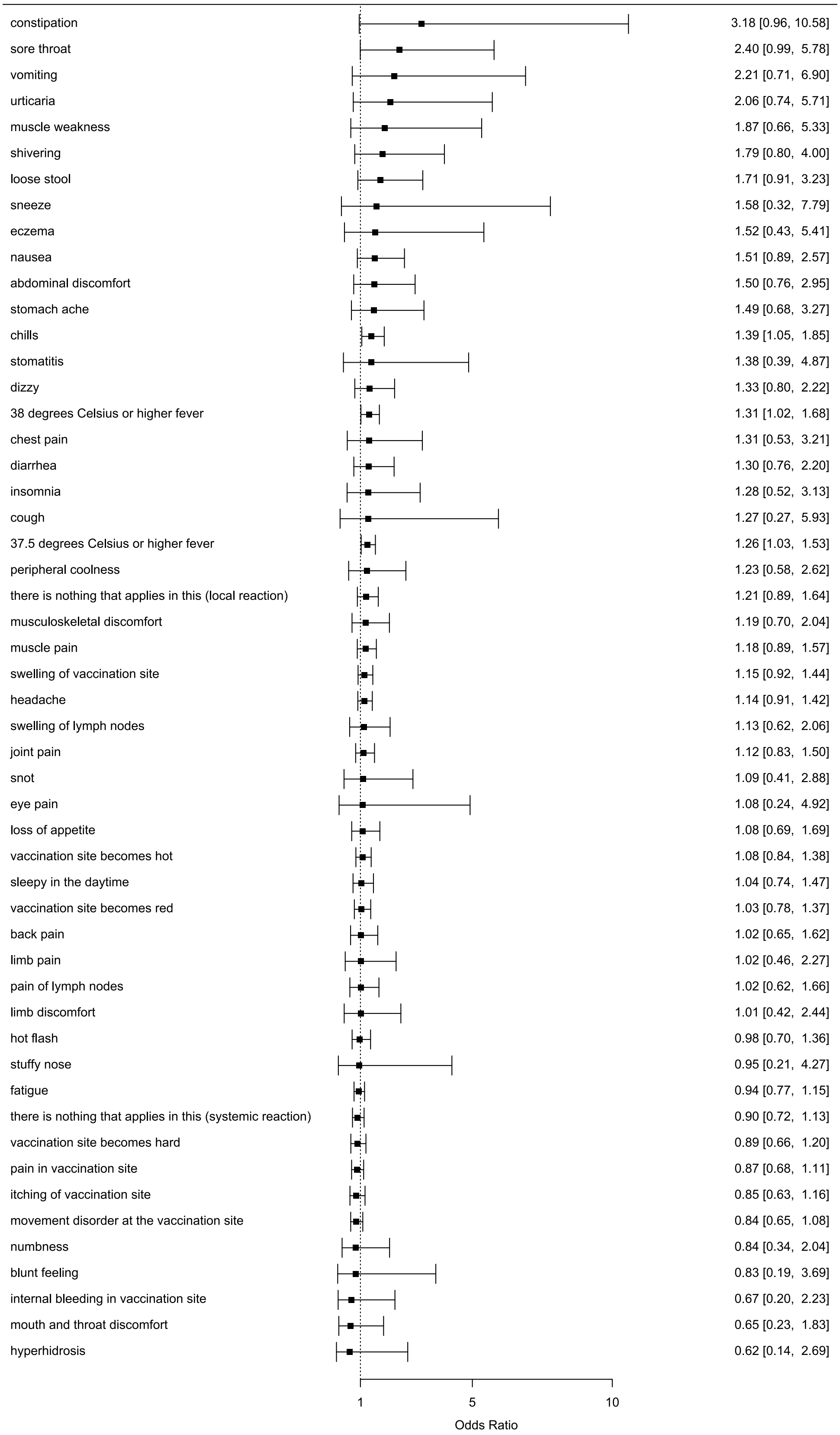

HLA-DQA1*03:01 at 1st dose of mRNA-1273 vaccine

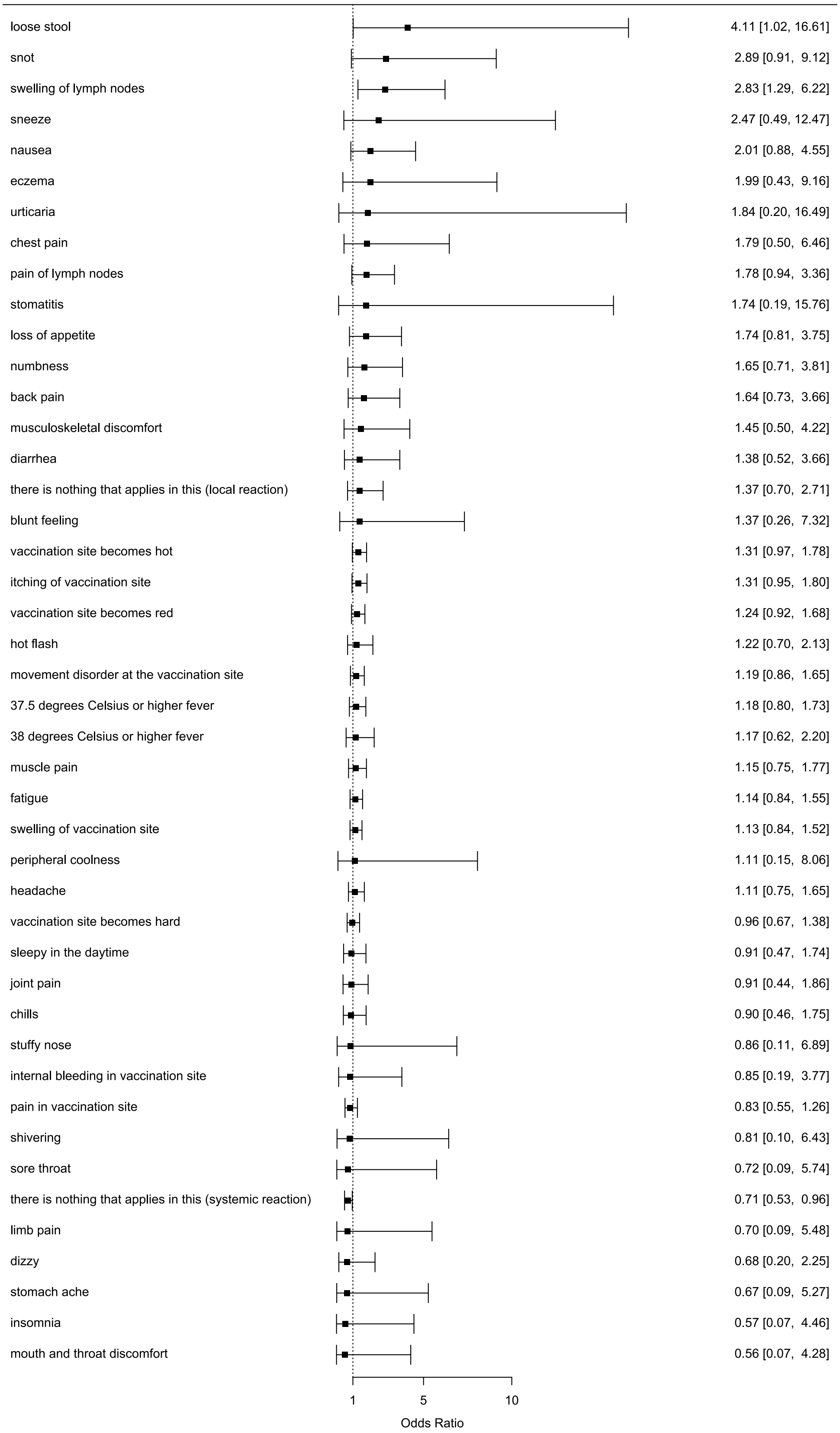

HLA-DQA1*03:01 at 2nd dose of mRNA-1273 vaccine

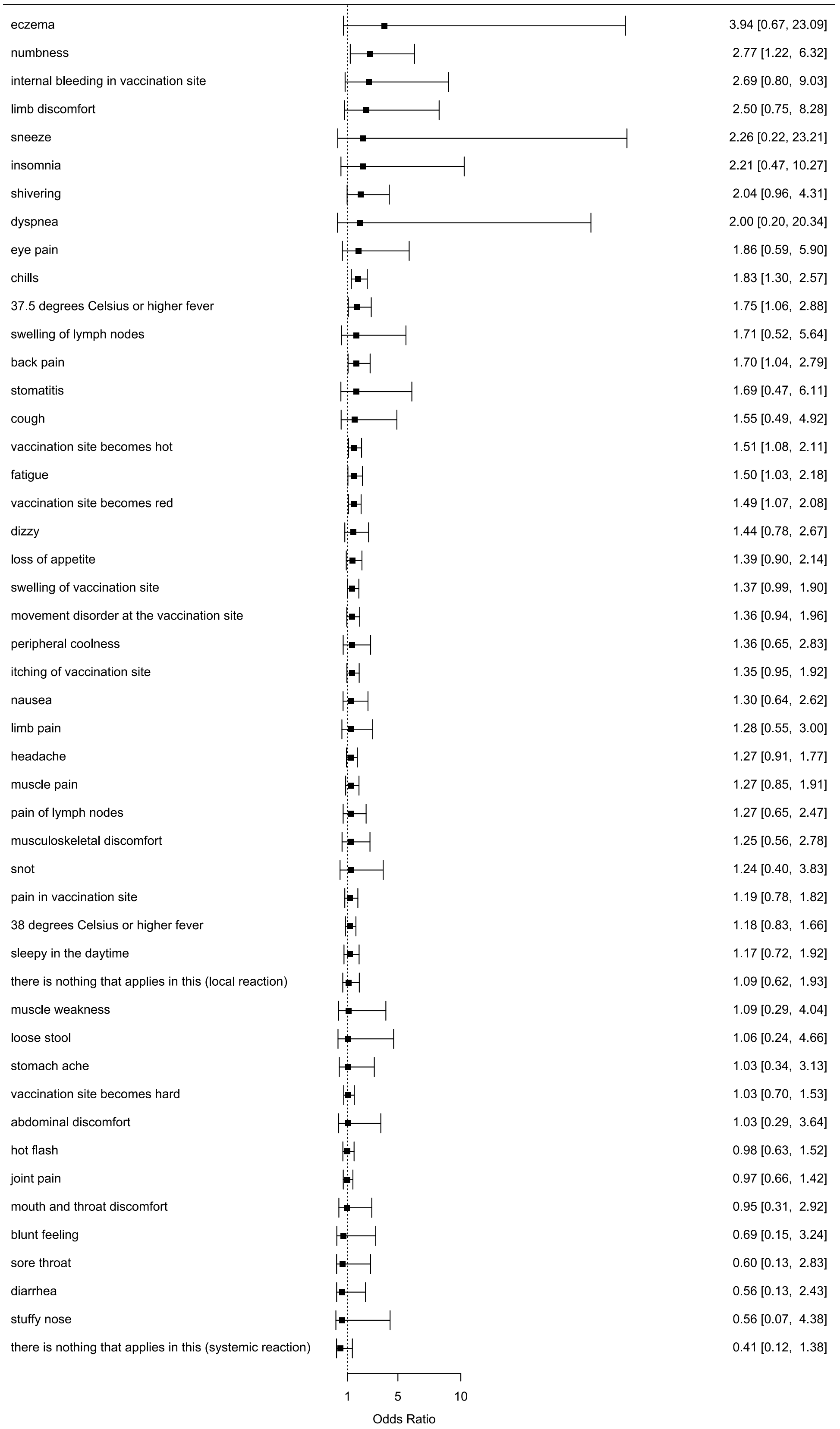

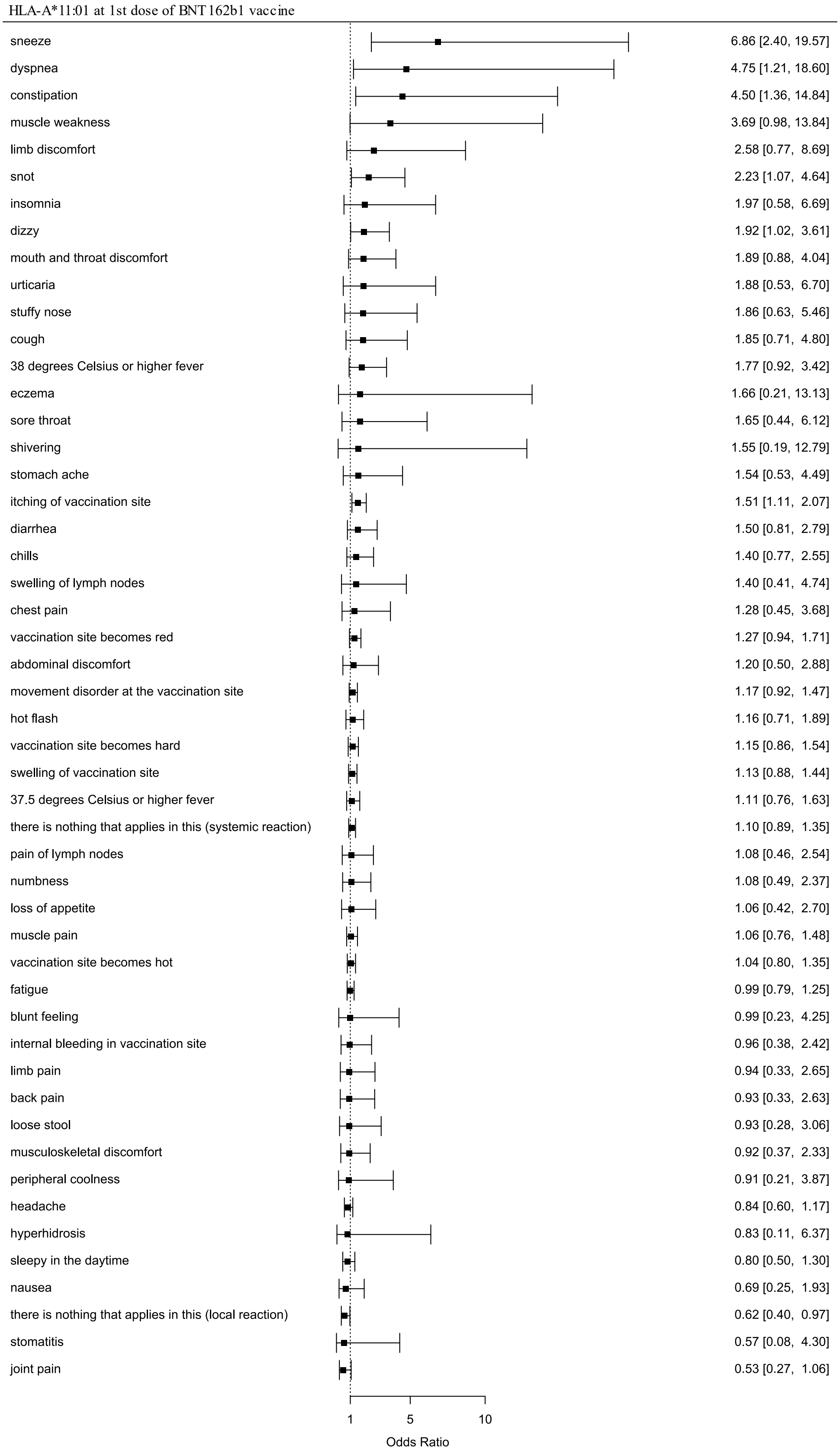
HLA-A*11:01 at 1st dose of BNT162b1 vaccine
sneeze 6.86 [2.40, 19.57]
dyspnea 4.75 [1.21, 18.60]
constipation 4.50 [1.36, 14.84]
muscle weakness 3.69 [0.98, 13.84]
limb discomfort 2.58 [0.77, 8.69]
snot 2.23 [1.07, 4.64]
insomnia 1.97 [0.58, 6.69]
dizzy 1.92 [1.02, 3.61]
mouth and throat discomfort 1.89 [0.88, 4.04]
urticaria 1.88 [0.53, 6.70]
stuffy nose 1.86 [0.63, 5.46]
cough 1.85 [0.71, 4.80]
38 degrees Celsius or higher fever 1.77 [0.92, 3.42]
eczema 1.66 [0.21, 13.13]
sore throat 1.65 [0.44, 6.12]
shivering 1.55 [0.19, 12.79]
stomach ache 1.54 [0.53, 4.49]
itching of vaccination site 1.51 [1.11, 2.07]
diarrhea 1.50 [0.81, 2.79]
chills 1.40 [0.77, 2.55]
swelling of lymph nodes 1.40 [0.41, 4.74]
chest pain 1.28 [0.45, 3.68]
vaccination site becomes red 1.27 [0.94, 1.71]
abdominal discomfort 1.20 [0.50, 2.88]
movement disorder at the vaccination site 1.17 [0.92, 1.47]
hot flash 1.16 [0.71, 1.89]
vaccination site becomes hard 1.15 [0.86, 1.54]
swelling of vaccination site 1.13 [0.88, 1.44]
37.5 degrees Celsius or higher fever 1.11 [0.76, 1.63]
there is nothing that applies in this (systemic reaction) 1.10 [0.89, 1.35]
pain of lymph nodes 1.08 [0.46, 2.54]
numbness 1.08 [0.49, 2.37]
loss of appetite 1.06 [0.42, 2.70]
muscle pain 1.06 [0.76, 1.48]
vaccination site becomes hot 1.04 [0.80, 1.35]
fatigue 0.99 [0.79, 1.25]
blunt feeling 0.99 [0.23, 4.25]
internal bleeding in vaccination site 0.96 [0.38, 2.42]
limb pain 0.94 [0.33, 2.65]
back pain 0.93 [0.33, 2.63]
loose stool 0.93 [0.28, 3.06]
musculoskeletal discomfort 0.92 [0.37, 2.33]
peripheral coolness 0.91 [0.21, 3.87]
headache 0.84 [0.60, 1.17]
hyperhidrosis 0.83 [0.11, 6.37]
sleepy in the daytime 0.80 [0.50, 1.30]
nausea 0.69 [0.25, 1.93]
there is nothing that applies in this (local reaction) 0.62 [0.40, 0.97]
stomatitis 0.57 [0.08, 4.30]
joint pain 0.53 [0.27, 1.06]
1
5
10
Odds Ratio

HLA-A*11:01 at 2nd dose of BNT162b1 vaccine

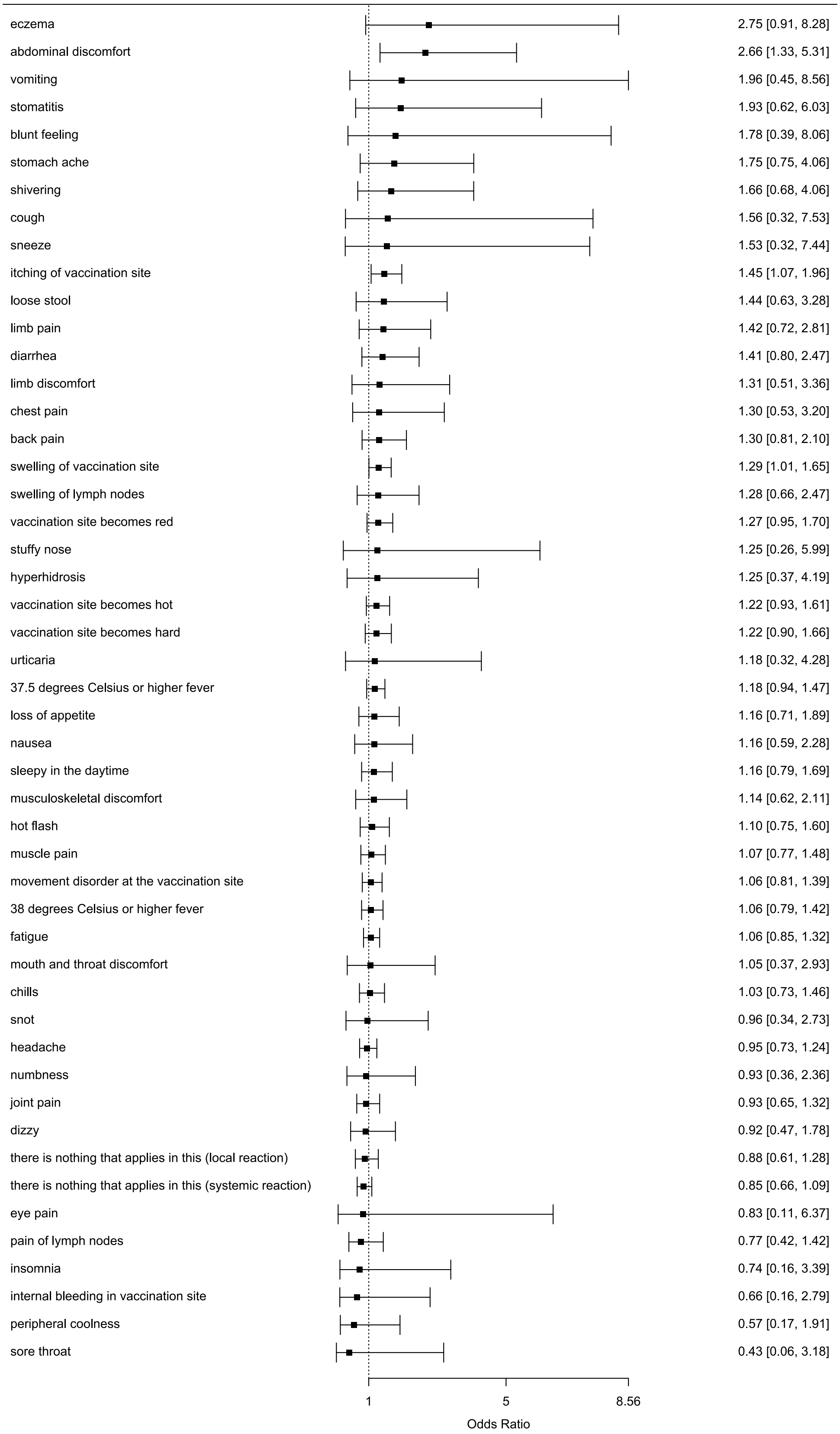

HLA-A*11:01 at 1st dose of mRNA-1273 vaccine

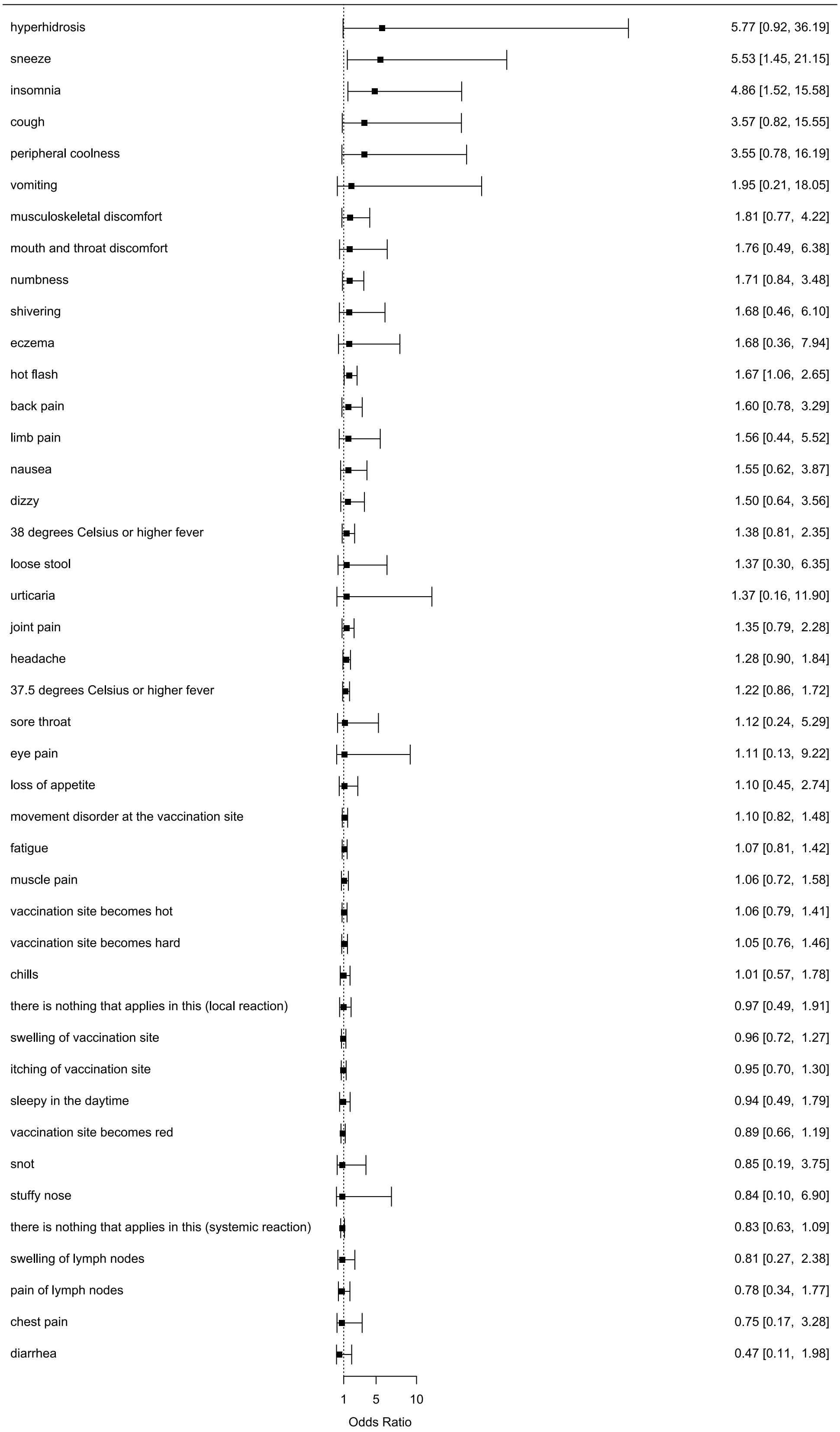

HLA-A*11:01 at 2nd dose of mRNA-1273 vaccine

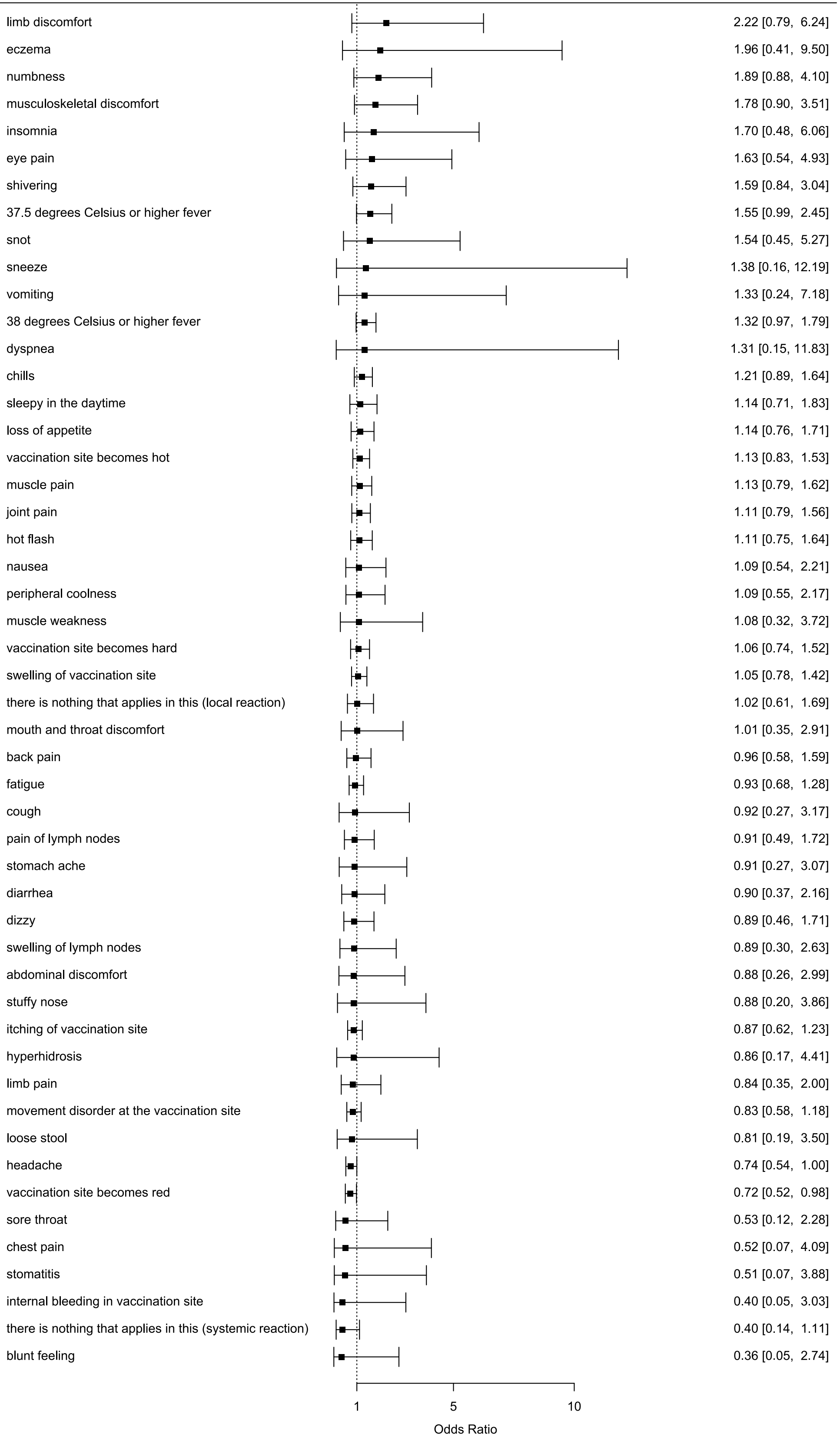

Supplementary Figure 2. Odds ratios and 95% confidence intervals of the two reliable HLA alleles (HLA-DQA1*03:01 and HLA-A*11:01) for the occurrence of all adverse events
